# Metabolic plasticity and optimal redox homeostasis are essential for efficient metastatic colonization

**DOI:** 10.1016/j.molmet.2026.102382

**Published:** 2026-05-20

**Authors:** Ece Grace, Deyu Zou, Romy Böttcher-Loschinski, Martin Böttcher, Harald Schuhwerk, Yussuf Hajjaj, Annemarie Schwab, Simon Brandt, Ana Clavel Ezquerra, Witold Szymanski, Johannes Graumann, Philipp Arnold, Renato Liguori, Fulvia Ferrazzi, Constantin P. Krempe, L.M. Nascentes Melo, Gabriele Allies, Sven W. Meckelmann, Dirk Mielenz, Simone Brabletz, Dimitrios Mougiakakos, Alpaslan Tasdogan, Thomas Brabletz, Marc P. Stemmler

**Affiliations:** 1 Department of Experimental Medicine 1, Nikolaus-Fiebiger Center for Molecular Medicine, Friedrich-Alexander University of Erlangen-Nürnberg (FAU), Erlangen, Germany; 2 Department of Hematology, Oncology and Cell Therapy, Otto-von-Guericke-University of Magdeburg, Magdeburg, Germany; 3 Magdeburg Centre for Cell and Immune Therapy (MAZI), Medical Faculty, Otto-von-Guericke University, Magdeburg, Germany; 4 Healthcampus Immunology, Inflammation and Infectiology (GC-I3), Medical Faculty, Otto-von-Guericke University, Magdeburg, Germany; 5 Department of Dermatology, University Hospital Regensburg, Regensburg, Germany; 6 Institute of Translational Proteomics & Core Facility Translational Proteomics, Philipps-Universität Marburg, Marburg, Germany; 7 Institute of Functional and Clinical Anatomy, University Hospital Erlangen, Friedrich-Alexander University of Erlangen-Nürnberg (FAU), Erlangen, Germany; 8 Department of Nephropathology, Institute of Pathology, University Hospital Erlangen, Friedrich-Alexander University of Erlangen-Nürnberg (FAU), Erlangen, Germany; 9 Institute of Pathology, University Hospital Erlangen, Friedrich-Alexander University of Erlangen-Nürnberg (FAU), Erlangen, Germany; 10 Comprehensive Cancer Center Erlangen-EMN (CCC ER-EMN), Bavarian Cancer Research Center (BZKF), Erlangen, Germany; 11 Department of Dermatology, University Hospital Essen, German Cancer Consortium (DKTK) and Research Alliance Ruhr, Research Center One Health, Campus Essen, Essen, Germany; 12 Applied Analytical Chemistry, University of Duisburg-Essen, Essen, Germany; 13 Department of Translational Immunology, Department of Internal Medicine 3, University Hospital Erlangen, Friedrich-Alexander University of Erlangen-Nürnberg (FAU), Erlangen, Germany; 14 Center for Health and Medical Prevention - CHAMP, Otto-von-Guericke University, Magdeburg, Germany

**Keywords:** Cancer, Metastasis, Metabolism, Redox balance, Cellular plasticity, Glycolysis, Mitochondria, Epithelial-to-mesenchymal transition, Ferroptosis, Pancreatic ductal adenocarcinoma (PDAC)

## Abstract

Cancer cells dynamically reprogram their metabolism to adapt to changing microenvironmental conditions during tumor growth and metastatic dissemination. Metastasis of solid tumors—the principal cause of cancer-related mortality—is often driven through activation of epithelial–mesenchymal transition (EMT), regulated by the transcription factor ZEB1, which is frequently upregulated during tumor progression. To investigate the role of metabolic plasticity in metastasis, we employed murine pancreatic ductal adenocarcinoma (PDAC) cell lines with distinct EMT states, ZEB1 expression and lung colonization capacities. Highly plastic epithelial-type cancer cells (KPCepi) efficiently colonize the lung, whereas *Zeb1*-deficient cancer cells (KPCZ) with compromised metabolic plasticity show markedly reduced colonization, correlated with absent glycolytic reserve, mitochondrial dysfunction, and reduced anti-oxidant metabolite levels. Interestingly, mesenchymal-type cancer cells (KPCmes) also exhibit poor lung colonization despite retaining normal glycolytic capacity and a high proportion of functional mitochondria; however, similar to KPCZ cells, they display diminished levels of detoxifying metabolites. Low metastatic capacity correlates with increased susceptibility to ferroptosis even in epithelial-type KPCZ cells, indicating a limited ability to counteract reactive oxygen species under stress. Together, these findings demonstrate that metabolic plasticity and redox homeostasis are essential prerequisites for efficient lung colonization. Thus, concurrent targeting of metabolic adaptability and redox buffering may represent a promising strategy to prevent metastasis in aggressive PDAC tumors.

## Highlights

• Metabolic adaptability of PDAC cells correlates with metastatic colonization efficacy.
• *Zeb1*-deficient and mesenchymal-type cells show reduced lung colonization capacity.
• *Zeb1* deficiency impairs glycolytic reserve, mitochondria and anti-oxidant levels.
• Low metastatic capacity correlates with increased susceptibility to ferroptosis.

## 1 Introduction

Cancer progression is characterized by continuously changing microenvironmental conditions, including nutrient availability and oxygen levels, to which tumor cells need to constantly adapt [1,2]. This phenomenon is most clearly illustrated during metastasis, where invasive cancer cells intravasate into the bloodstream and subsequently disseminate to distant organs such as the lungs or liver, where they encounter markedly distinct microenvironments [[3], [4], [5], [6]]. To withstand these environmental changes and ensure survival, successful colonization, and rapid proliferation, tumor cells require a high degree of metabolic adaptability. This aberrant metabolic adaptability encompasses flexibility—the capacity to use diverse metabolic substrates—and plasticity—the capacity to process these substrates through alternative pathways [7]. This includes the ability to adapt glycolysis and the TCA cycle to foster aerobic and anaerobic ATP production and to switch between glycolysis and oxidative phosphorylation, as well as to exploit alternative nutrient sources such as glutamine or lipids [2,8,9]. Beyond energy production, these metabolic adaptations also need to protect cancer cells from excessive oxidative damage and maintenance of redox homeostasis, which is particularly challenged during metastatic dissemination [10]. The capacity to regenerate reducing equivalents, such as NADPH, and to sustain antioxidant systems including glutathione, is therefore a critical determinant of metastatic fitness [[11], [12], [13], [14], [15], [16]].

Although metastasis is the primary cause of cancer-related mortality, it represents a complex multistep process that is highly inefficient. Only a small fraction of circulating tumor cells manages to survive the diverse environmental stresses encountered during dissemination to the lung or other organs. This underscores the critical importance of adaptive capacity and acquisition of phenotypic plasticity [[17], [18], [19], [20]]. One of the central processes enabling cell plasticity and orchestrating metastasis is the epithelial–mesenchymal transition (EMT) program [[21], [22], [23], [24], [25]]. Induced by environmental signals, like TGFβ, it is activated intracellularly by ZEB1 and other core EMT transcription factors [21,23,24,26]. Loss of *Zeb1* attenuates a TGFβ-induced cellular response and reduces both metabolic and phenotypic adaptability, emphasizing its pivotal role in maintaining tumor cell plasticity [27].

To examine how regulation of metabolic plasticity promotes efficient lung colonization and to delineate the role of ZEB1 in cellular and metabolic plasticity, we utilized pancreatic cancer cell lines derived from Pd*x*1-Cre; *Kras*^*G12D*^; *Tp53*^*R172H*^ (KPC) mouse tumors with either wild-type or conditionally inactivated *Zeb1* [27]. We show that cellular plasticity and phenotypic states across the EMT spectrum are characterized by distinct metabolic profiles, in which specific configurations of glycolysis, TCA cycle, mitochondrial function, and reactive oxygen species (ROS) detoxification correlate with lung colonization capacity. Furthermore, we provide evidence that perturbations in any of these pathways are associated with impaired metastatic colonization.

## 2 Materials and methods

### 2.1 Animal experiments

Animal husbandry and all experiments were performed according to the European Animal Welfare laws and guidelines. The protocols were approved by the committee on ethics of animal experiments of Bavaria (Regierung von Unterfranken, Würzburg; TS-18/14, 55.2-DMS-2532-2-59). Power analysis was used to calculate the sample size required for animal experiments. Animals were kept on a 12:12 h light–dark cycle and provided with food and water ad libitum in the animal facilities of the Friedrich-Alexander University of Erlangen-Nürnberg.

Lung colonization capacity was assessed by tail-vein injection into syngeneic C57BL/6N mice. Cells were trypsinized (Gibco, 25200056) and resuspended in PBS (Gibco, 14190094). 2 × 10^5^ cells in 100 μl were injected into the lateral tail vein using a 27G needle. Mice were euthanized 18 days post-injection, and lungs were isolated. Lungs were fixed in 4% PFA/PBS overnight at 4 °C, paraffin embedded, sectioned at 3–4 μm and subjected to haematoxylin/eosin (H&E) staining as described previously [27]. Metastatic lesions were quantified in two independent sections per lung separated by at least 200 μm. Each cell line was injected into three mice.

### 2.2 Primary cell lines

KPCepi (KPC661, KP792 and KPC865), KPCmes (KPC550, KPC701 and KPC827) and KPCZ (KPCZ346, KPCZ426 and KPCZ519) cell lines were derived from PDAC tumors of KPC and *Zeb1*-deficient KPC mice, described before [27]. Cells were cultured in DMEM (Gibco, 31966021)/10% FBS (Gibco, 10438026) at 37 °C/5% CO_2_ in a humidified incubator and passaged as described previously [27]. With the exception of proteomic analyses, two cell lines per phenotype/genotype were used for all other experiments (KPCepi, KPC661, KP792; KPCmes, KPC550, KPC701; KPCZ, KPCZ346, KPCZ426).

### 2.3 Seahorse XF bioenergetic assay

The bioenergetic profiles of KPCepi, KPCmes and KPCZ cell lines were analyzed using an XFe96 Extracellular Flux Analyzer (Agilent Seahorse XFe96 analyzer, RRID: SCR_019545). Cells were seeded in Seahorse XF96 cell-culture microplates at a density of 15,000 cells per well and cultured for 18 h in DMEM/10% FCS. One hour before measurement, medium was exchanged with Seahorse assay medium and cells were incubated at 37 °C in a CO_2_-free atmosphere. Glycolytic parameters were determined using the Seahorse Glycolysis Stress Test (GST). Basal extracellular acidification rate (ECAR), indicative of glycolytic activity was measured under glucose-free conditions. ECAR was then recorded following glucose supplementation (10 mM) to calculate the glycolytic rate. Glycolytic capacity and glycolytic reserve were determined after sequential addition of oligomycin (1 μM; Sigma, 75351) to inhibit mitochondrial ATP synthesis and 2-deoxyglucose (2DG; 100 mM; Sigma, D6134) to inhibit hexokinase activity. Mitochondrial respiration was assessed using the Seahorse Mito Stress Test (MST). Basal oxygen consumption rate (OCR) was recorded, followed by the sequential addition of oligomycin (1 μM; Sigma, 75351), carbonyl cyanide 4-(trifluoromethoxy) phenylhydrazone (FCCP; 1 μM; Sigma, C2920), and a combination of antimycin A (3 μM; Sigma, A8674) and rotenone (3 μM; Sigma, R8875). Values were normalized in the XF Wave Software using total protein content as measured by Pierce BCA Protein Assay Kit (Thermo Fisher) immediately after the run. These data were used to calculate basal respiration, maximal respiration, and ATP-linked respiration. All experiments were performed in pentaplicates.

### 2.4 NADH/NAD^+^, NADPH/NADP^+^ and GSH/GSSG quantification

KPCepi, KPCmes and KPCZ cells were seeded in 10,000 cells per well in 96-wells (Greiner Bio-One, 655075) for the GSH/GSSG assay. 40,000 cells in 50 μl PBS were divided into two wells of 96-wells (Greiner Bio-One, 655075) for NADH/NAD^+^ and NADPH/NADP^+^ quantifications. NAD^+^ and NADH levels were measured using the NAD/NADH-Glo™ Assay (Promega, G9071), NADP^+^ and NADPH levels were measured using the NADP/NADPH-Glo™ Assay (Promega, G9081) and reduced and oxidized glutathione (GSH and GSSG) levels were determined using the GSH/GSSG-Glo™ Assay (Promega, V6611), following the manufacturer’s instructions.

Standard curves were generated using purified NAD (Sigma–Aldrich, 8285), NADH (Sigma–Aldrich, 6785), NADP monosodium salt (Santa Cruz Biotechnology, sc-202724), NADPH tetrasodium salt (Santa Cruz Biotechnology, sc-202725A) and GSH (provided in the Promega kit), which were resuspended in the same buffer as the experimental samples from each assay. The absolute amounts of NAD^+^, NADH, NADP^+^, NADPH, GSH, and GSSG for KPCepi, KPCmes and KPCZ cells were determined from individual standard curves. Luminescence was measured using a Centro XS^3^ LB 960 microplate luminometer (Berthold Centro LB 960 Microplate Luminometer; RRID:SCR_026977).

### 2.5 Quantification of hexokinase (HK), phosphofructokinase (PFK) and glucose-6-phosphate dehydrogenase (G6PD) activities

For HK activity measurements cells were resuspended in ice-cold HK assay buffer after trypsinization and 5 μl cell suspension containing 2.5 × 10^4^ cells were transferred into 96-wells (Sigma–Aldrich, TPP92096) and analyzed by the hexokinase colorimetric assay kit (Sigma–Aldrich, MAK091) according to the manufacturer’s instructions. Absorbance was measured in a FLUOstar Omega plate reader (BMG Labtech FLUOstar Omega; RRID: SCR_025024) and HK activity was calculated.

For PFK activity measurements 1,250 cells were resuspended in ice-cold PFK assay buffer and processed according to the PFK activity colorimetric assay kit (Sigma–Aldrich, MAK093) protocol. Absorbance was measured in a FLUOstar Omega plate reader (BMG Labtech FLUOstar Omega; RRID: SCR_025024) and PFK activity was calculated.

For G6PD activity measurements 3 × 10^6^ cells were collected by centrifugation and cell pellets were resuspended in 1 ml of cold PBS, sonicated on ice and centrifuged at 10,000 g for 10 min at 4 °C. Supernatants were removed and cell pellets resupended in the assay buffer/cofactor/enzyme mix and transferred to 96-wells (revvity, 6055300) for determining GDPD activity according to the manufacturer's instructions (Cayman Chemical, 700300) protocol. Luminescence was measured using a Centro XS^3^ LB 960 microplate luminometer (Berthold Centro LB 960 Microplate Luminometer; RRID:SCR_026977) and G6PD was calculated.

### 2.6 Isotope tracing analysis by GC–MS

For gas chromatography–tandem mass spectrometry (GC–MS) analysis, 6 × 10^5^ KPCepi, KPCmes and KPCZ cells were seeded in 10-cm plates to allow collection of cell pellets at multiple time points (0 min, 30 min, 1 h, 2 h) for assessment of isotope labeling over time. For the 24-h time point, 3 × 10^5^ cells were plated. Cells were cultured in DMEM (Gibco, A1443001) supplemented with 10% FBS (Gibco, 10438026), 10 mM glucose (Gibco, A2494001), 1 mM GlutaMAX (Gibco, 35050038), and 1 mM pyruvate (Gibco, 11360039). Twenty-four hours prior to isotope labeling, the medium was replaced with the same formulation lacking FBS to synchronize the cell cycle. At 80–100% confluence, cells were washed three times with PBS and incubated with experimental medium consisting of DMEM supplemented with 10 mM [U–^13^C]-glucose (Cambridge Isotope Laboratories, CLM-1396), 1 mM GlutaMAX, 1 mM pyruvate, and 10% FBS. Cells were incubated for the indicated time points at 37 °C in a humidified incubator with 5% CO_2_. Isotope labeling was terminated by placing the plates on ice, followed by three washes with ice-cold PBS. After removal of PBS, 600 μl of 80% ice-cold methanol (Carl Roth, 0082.3) was added, and cells were scraped, transferred to 1.5-ml Eppendorf tubes, and snap-frozen in liquid nitrogen. Cell extracts were vortexed and subjected to three freeze–thaw cycles in liquid nitrogen. Following centrifugation at 15,000 rpm for 15 min at 4 °C, the supernatant was collected, lyophilized using a SpeedVac (Thermo Fisher) and resuspended in 30 μl of anhydrous pyridine containing methoxyamine hydrochloride (10 mg/ml). The resulting solution was transferred to pre-prepared GC–MS autoinjector vials containing 70 μl N-tert-butyldimethylsilyl-N-methyltrifluoroacetamide (MTBSTFA) for derivatization of polar metabolites. Samples were incubated at 70 °C for 1 h, after which 1-μl aliquots were injected for analysis. GC–MS measurements were performed using an Agilent 8890 gas chromatograph (Agilent 8890 GC System; RRID:SCR_019459) coupled to an Agilent 5977B mass selective detector (Agilent 5977B GC/MSD; RRID:SCR_019420). Observed isotopologue distributions were corrected for natural isotope abundance [28].

### 2.7 Flow cytometry

5 × 10^5^ cells (KPCepi, KPCmes and KPCZ cells) were resuspended in PBS and transferred to FACS tubes and centrifuged at 300 g for 5 min. Cell pellets were resuspended in 100 μl 10 nM MitoTracker Green (Invitrogen, M7514) or 10 nM MitoTracker Deep Red (Invitrogen, M22426) and incubated for 30 min at 37 °C. Samples were washed by adding cell culture media. Cells were centrifuged and resuspended in 300 μl washing buffer (PBS+0.25% PBA). Data acquisition was performed in a CytoFlex analyzer (Beckman Coulter CytoFLEX S V4–B2–Y4-R3 Flow Cytometer; RRID:SCR_027083) and analyzed using CytExpert (CytExpert; RRID:SCR_017217) and Kaluza softwares (Kaluza; RRID:SCR_016182).

### 2.8 RNA expression analysis by qRT-PCR

Total RNA was isolated using the RNeasy Plus Mini Kit (QIAGEN, 74136) following the manufacturer’s instructions. Briefly, KPCepi, KPCmes and KPCZ cells were washed twice with 5 ml PBS and lysed in 350 μl RLT Plus buffer. 1 μg of total RNA was reversely transcribed into cDNA using the RevertAid First Strand cDNA Synthesis Kit (Thermo Fisher, K1622) according to the manufacturer’s protocol. Quantitative reverse transcription PCR (qRT-PCR) was performed in triplicates using cDNA of 7.5 ng total RNA in 10 μl volume with gene-specific primers, TaqMan Universal Master Mix II (Thermo Fisher Scientific, 4440044) and the Universal Probe Library (UPL) system (Roche, 04869877001) in 384-well plates with a LightCycler 480 II instrument (Roche LightCycler 480 Real Time PCR System; RRID:SCR_018626). Alternatively, mRNA expression levels were quantified using the Power SYBR Green PCR Master Mix (Applied biosystems, 4367659) without UPL. Values were normalized to *Gapdh*. Primer sequences are listed in Supplementary Table 3.

### 2.9 Protein mass spectrometric analysis

For proteomic analysis, KPCepi, KPCmes and KPCZ cells were harvested from 10 cm plates and processed as described in the Supplementary methods. Using an SP3-based workflow [29] resulting peptides were analyzed by LC–MS/MS on a Bruker timsTOF Pro Instrument (Bruker timsTOF Pro mass spectrometer; RRID:SCR_026544) and processed using DIA-NN (Data-Independent Acquisition by Neural Networks, version 1.8.1 Academia) [30] in a library-free mode against the mouse Uniprot database (RRID: SCR_002380, August 2022, reviewed) database. Pathway enrichment analysis was conducted using Gene Set Enrichment Analysis (GSEA) [31] as implemented in the gseKEGG() function of the clusterProfiler R package (clusterProfiler; RRID:SCR_016884, v4.12.6) [32], with KEGG pathways considered significant at an adjusted *P*-value <0.05. Analyses were performed across nine biological replicates representing three independent cell lines per condition. See Supplementary methods for details.

### 2.10 Western blot analysis

For whole-cell protein analysis, cells at 50–70% confluence were lysed in ice-cold RIPA buffer (150 mM NaCl, 50 mM Tris–HCl (pH 8.0), 0.5% (w/v) sodium deoxycholate, 0.1% (v/v) SDS, 1% (v/v) NP-40, 1 mM PMSF, 1 × complete protease inhibitor cocktail (Roche, 4693132001), 1 × PhosphoStop (Roche, 4906837001)). Protein concentrations were quantified using the BCA Protein Assay kit (Thermo Fisher, NEL105001EA) following the manufacturer’s protocol. Equal amounts of protein were separated by SDS–PAGE and transferred to nitrocellulose membranes using wet transfer. Membranes were incubated with primary antibodies overnight at 4 °C, followed by incubation with appropriate HRP-coupled secondary antibodies for 1 h at room temperature. Protein signals were detected using Western Lightning Plus ECL reagent (PerkinElmer) or Clarity Western ECL Substrate (Bio-Rad, 1705061) and visualized with a ChemiDoc MP Imaging System (Bio-Rad) using Image Lab software (version 6.1). . The following antibodies were used: rabbit anti-ZEB1 (1:2,000, Sigma–Aldrich, HPA027524), mouse anti-E-cadherin (1:15,000, BD Transduction Laboratories, 610182), rabbit anti-HK2 (1:1; 000, Cell Signaling, 2867), rabbit anti-PFKP (1:1,000, Proteintech, 13389-1-AP), rabbit anti-PFKM (1:1,000, Proteintech, 55028-1-AP), rabbit anti-PFKFB3 (1:1,000, Proteintech, 13763-1-AP), rabbit anti-PKM2 (1:1,000, Cell Signaling, 4053), rabbit anti-G6PD (1:1,000, Proteintech, 25413-1-AP), rabbit anti-ME1 (1:2,000, Proteintech, 16619-1-AP), rabbit anti-IDH1 (1:2,000, Proteintech, 12332-1-AP), rabbit anti 4-HNE (1:1,000, Abcam, ab46545), rabbit anti-GPX4 (1:2,000, Abcam, AB125066-1001), mouse anti-β-actin (1:10,000, Sigma–Aldrich, A5441). For detection of ferroptosis markers cells were treated with 1 μM ML210 (MedChem Express, HY-100003) for 4 h.

### 2.11 ChIP-seq analysis and luciferase reporter assays

For identification of ZEB1 binding to gene loci of metabolic enzymes, publicly available human ZEB1 ChIP-seq datasets were retrieved from the Cistrome Data Browser (Cistrome DB) in bigWig format. The processed genomic coverage tracks were subsequently imported into the Integrative Genomics Viewer (IGV, version 2.16.0) for visualization and assessment of ZEB1 binding enrichment across the Hg38 reference genome [[33], [34], [35], [36], [37], [38]].

### 2.12 siRNA transfection

8 × 10^4^ KPCepi (KPC661, KPC792) cells were seeded in 6-wells 24 h before transfection. For siRNA-mediated *Zeb1* knockdown, siRNAs were obtained from Ambion (Silencer Select siRNAs: ZEB1, 5′-GGCUGUAGAUGGUAACGUATTT-3′ and Silencer Select Negative Control #1 siRNA (4390844)) and transfected at a final concentration of 50 nM with Lipofectamine RNAiMAX transfection reagent (Thermo Fisher, 13778) according to the manufacturer’s instructions. Cells were harvested 48 h post transfection.

### 2.13 Transmission electron microscopy

4 × 10^5^ KPCepi cells, 3 × 10^5^ KPCmes and 2 × 10^5^ KPCZ cells were seeded per well of a 6-well plate. Once the cells reached 60% confluence, media was replaced by 2 ml ITO-buffer (2.5% PFA, 2.5% glutaraldehyde, 0.1% picric acid in 0.1 M cacodylate buffer, pH 7.3) for fixation. Plates were incubated for 30 min at room temperature, before the ITO-buffer was exchanged to 2 ml of fresh solution and plates were kept at 4 °C until further processing. Fixation with osmium-tetroxide, embedding in raisin, trimming and thin-sectioning followed standard procedures [39] and was performed in the 6-well format with the following exceptions. Samples were embedded into epon (Roth, 8619.2) and a cold shock of liquid nitrogen was used to detach the polymerized epon containing the cells from the plastic support layer of the 6-well. After trimming to obtain the areas of interest, the protocol followed standard procedures as described [40]. Mitochondria were classified into eight subcategories based on cristae integrity, cristae density, cristae spacing, cristae circularity and overall mitochondrial morphology as described [41]. Categories 1 and 8 were the extremes which were represented in Suppl. Fig. 6A. Categories 1–3 represented proper mitochondria with intact cristae and low inter-cristae spacing, whereas categories 4–8 showed a gradual increase in cristae spacing and mitochondrial swelling. Intermediate categories reflected progressive structural deterioration, with category 8 displaying severely swollen mitochondria, sparse cristae, and markedly increased inter-cristae distance.

### 2.14 Isolation of genomic DNA and mitochondrial DNA quantification

8 × 10^5^ KPCepi cells, 4 × 10^5^ KPCmes and 4 × 10^5^ KPCZ cells were seeded per well of a 6-well plate. The next day cells were washed with 500 μl of PBS, scraped and centrifuged at 1000 rpm for 5 min. Cell pellets were resuspended in PBS and DNA was isolated by using the QIAamp DNA blood mini kit (Qiagen, 51104) according to the manufacturer’s instructions. Mitochondrial and genomic DNA was analyzed by qPCR using gene-specific primers (Supplementary Table 3) and the SYBR Green PCR Master Mix in the LightCycler 480 II instrument (Roche LightCycler 480 Real Time PCR System; RRID:SCR_018626). Samples were analyzed in triplicates for *mt-Tl1* and *mt-Rnr2* mtDNA copy numbers and normalized to nuclear genomic DNA by *Gapdh*.

### 2.15 Cell viability assay

To determine cell viability, 2,000 cells (KPCepi, KPCmes and KPCZ cells) were seeded per well of a 96-well plate. After 24 h, cells were treated with vehicle or ML210 compound (MedChem Express, HY-100003) at concentrations of 10 μM, 5 μM, 2.5 μM, 1.25 μM, 0.63 μM, 0.31 μM, 0.16 μM, 0.08 μM and 0.04 μM for 69 h. DMSO served as solvent control. Cell viability was assessed by the confluence matrix using the live-cell imaging device Incucyte S3 (Sartorius IncuCyte S3 Live Cell Analysis System; RRID:SCR_023147). Relative confluence was calculated as described previously [42].

### 2.16 Lipid peroxidation assay

Lipid peroxidation was assessed using BODIPY 581/591C11 (Thermo Fisher, D3861). For each treatment condition 1 × 10^5^ KPCepi (KPC661, KPC792), KPCmes (KPC550, KPC701) and KPCZ (KPCZ346, KPCZ426) cells were seeded into 6-wells. The following day, cells were treated with DMSO (vehicle control) or 0.2 μM ML210 (MedChem Express, HY-100003) with or without 10 μM Ferrostatin-1 (MedChem Express, HY-100579) for 2 h or 1 μM ML210 with or without 10 μM Ferrostatin-1 for 1 h. Cells were subsequently stained with 1 μM BODIPY 581/591C11 diluted in Fluorobrite DMEM (Thermo Fisher, A1896701) supplemented with 1% GlutaMAX (Gibco, 35050038) for 30 min at 37 °C, 5% CO_2_. Cells were harvested, centrifuged (1,200 rpm, 5 min), resuspended in PBS + Zombie NIR viability dye (Biolegend, 423105) and incubated for 10 min at RT. After washing in PBS/2% FBS/0.5 mM EDTA flow cytometric analysis was performed using the CytoFlex analyzer (Beckman Coulter CytoFLEX S V4–B2–Y4-R3 Flow Cytometer; RRID:SCR_027083). After gating for alive (Zombie NIR negative), single cells (SSC-A vs. SSC-H), PE (reduced probe) and FITC (oxidized probe) fluorescence were recorded. Oxidation of BODIPY C11 581/591 was determined as the ratio of green to total (green + red) fluorescence, normalized to each corresponding DMSO control.

### 2.17 Statistical analysis

Statistical analysis was performed using GraphPad Prism software (GraphPad Prism; RRID:SCR_002798, version 10). The n-numbers represent the number of biological replicates in each group as indicated for each experiment. Statistical significance was assessed as indicated, depending on assay-specific sampling as well as the type and distribution of the obtained data. *P* values < 0.05 were considered statistically significant.

## 3 Results

### 3.1 Altered glycolysis and loss of glycolytic reserve correlate with poor lung colonization

**Figure 1:**
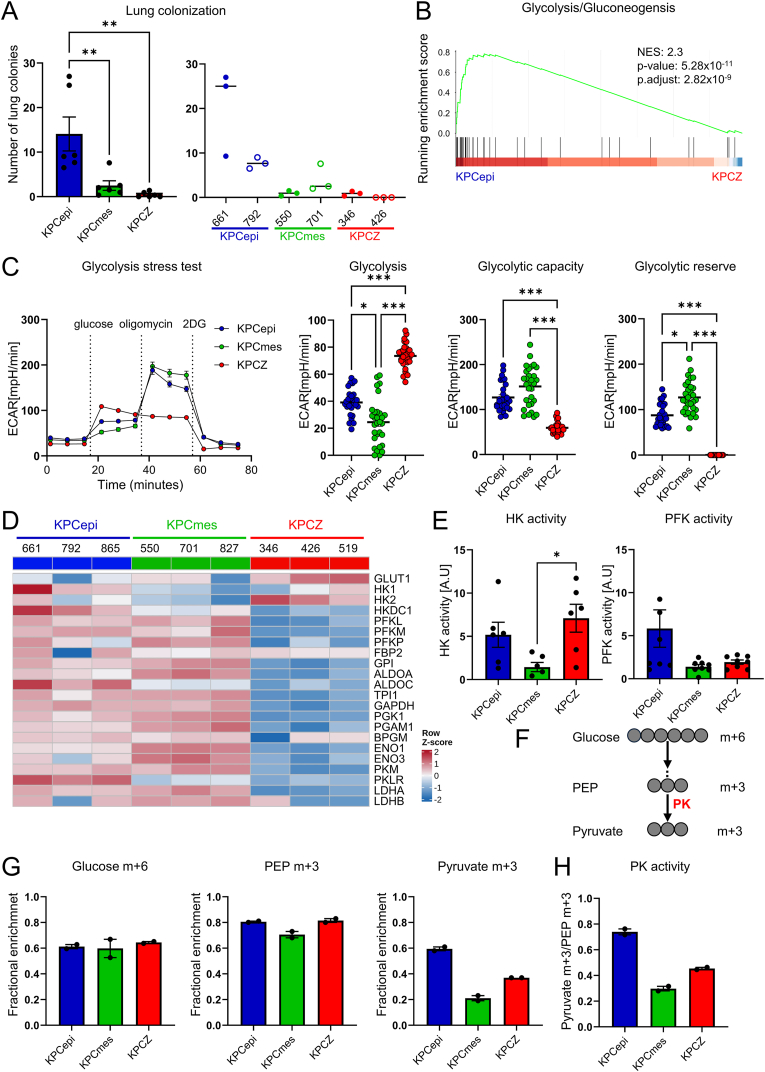
**Glycolytic flexibility, a hallmark of metabolic plasticity, correlates with the lung colonization capacity of pancreatic ductal adenocarcinoma (PDAC) cell lines.** **A.** Quantification of lung colonies three weeks after tail vein injection of KPC tumor cells into isogenic mice grouped by phenotype/genotype (left) and for individual cell lines (right). *n* = 6 biological replicates, 3 mice per line (KPCepi (KPC661, KPC792), KPCmes (KPC550, KPC701) and KPCZ (KPCZ346, KPCZ426). **B.** Gene set enrichment analysis (GSEA) of the KEGG glycolysis/gluconeogenesis pathway showing enrichment based on differential protein expression quantified by mass spectrometry comparing KPCepi vs. KPCZ cells. **C.** Glycolysis stress test run in a Seahorse instrument measuring extracellular acidification rate (ECAR) upon addition of glucose, oligomycin and 2-deoxy-d-glucose (2-DG) and calculating rates of glycolysis, glycolytic capacity and glycolytic reserve. *n* = 6 biological replicates, 2 cell lines for each phenotype/genotype (KPCepi, KPCmes, KPCZ), run in 5 technical replicates. **D.** Heatmap of protein expression of cell lines as indicated showing relative abundance of key enzymes involved in glycolysis quantified by mass spectrometry and visualized as row z-score–scaled values. **E.** Quantitative analysis of enzymatic activities of rate limiting enzymes in glycolysis, hexokinase (HK) and phosphofructokinase (PFK). *n* = 6 and *n* = 8 respectively, 2 cell lines for each phenotype/genotype. **F.** Schematic representation of [U–^13^C]- glucose labeling and analyzed metabolites. Gray circles indicate number of [^13^C] carbon atoms in each metabolite. **G.** Isotope tracing of glycolysis-related metabolites in KPCepi, KPCmes and KPCZ cell lines by mass spectrometry 1 h after addition of [U–^13^C]-glucose. **H.** Pyruvate kinase (PK) activity calculated by the ratio of pyruvate m+3 and PEP m+3. *n* = 2 biological replicates, 2 cell lines for each phenotype/genotype. Data are represented as mean ± SEM. ∗*P* < 0.05, ∗∗*P* < 0.01, ∗∗∗*P* < 0.001 by Kruskal–Wallis test (C) or one-way ANOVA with Tukey’s test (A and E).

We previously showed that lung colonization, a critical step in the metastatic cascade, is highly dependent on the presence of *Zeb1* [27]. Pancreatic cancer cells, which we derived from the KPC mouse model, can be classified into epithelial (KPCepi; KPC661, KPC792, KPC865) and mesenchymal phenotypes (KPCmes; KPC550, KPC701, KPC827), while cells originating from *Zeb1*-deficient tumor cells are exclusively epithelial (KPCZ; KPCZ346, KPCZ426, KPCZ519) [27] (Suppl. Fig. 1, Supplementary Table 1). Following tail vein injection of two cell lines per phenotype/genotype, *Zeb1*-deficient tumor cells exhibited markedly reduced lung colonization efficiency. Notably, highly mesenchymal tumor cells showed a similarly pronounced impairment in lung metastatic capacity, as shown previously [27] (Figure 1A). To investigate the molecular basis underlying these differences, we performed proteomic analyses of all KPCepi, KPCmes, and KPCZ cells under steady-state culture conditions which revealed substantial differences in protein expression in all comparisons (Suppl. Fig. 2). KEGG pathway analysis revealed a significant downregulation of glycolytic enzymes in KPCZ cells compared with KPCepi cells (Figure 1B, Suppl. Fig. 3, Supplementary Tables 1 and 2), suggesting that altered glycolytic regulation may contribute to impaired lung colonization.

Functional analyses using metabolic flux assays (Seahorse) confirmed previous findings that ZEB1 depletion leads to a loss of glycolytic reserve and a marked reduction in glycolytic capacity. Despite exhibiting higher basal glycolytic rates as compared to KPCepi cells, KPCZ cells were unable to switch from oxidative phosphorylation to glycolysis, indicating a specific impairment of metabolic plasticity (Figure 1C), confirming previous findings [27]. Moreover, KPCmes cells exhibited a different metabolic response in the glucose stress test. Although they displayed comparable or even increased glycolytic capacity and reserve, their basal glycolysis rates were lower, and they required more time to upregulate glycolysis following glucose addition (Figure 1C).

Because efficient colonization and metastatic outgrowth depend on a high degree of metabolic adaptability, we examined whether alterations in the expression of core metabolic pathway proteins are associated with the reduction in lung colonization capacity of KPCZ and KPCmes cells. Proteomic analyses revealed a highly specific and coordinated reduction in the abundance of nearly all glycolytic enzymes upon loss of *Zeb1* (Figure 1D, Supplementary Table 1). In KPCZ cells, only the glucose transporter GLUT1 and hexokinase 2 (HK2) were upregulated. In contrast, KPCmes cells exhibited largely unchanged protein expression profiles relative to KPCepi cells, with the notable exceptions of reduced levels of hexokinase 1 (HK1), hexokinase 2 (HK2), aldolase C (ALDOC) and one of the two pyruvate kinase isoforms, pyruvate kinase liver and red blood cell (PKLR).

Given that many glycolytic enzymes are subject to allosteric regulation [43,44], we next assessed whether changes in protein abundance correlated with enzymatic activities. We analyzed the kinetics of two rate-liming glycolytic enzymes, HK and phosphofructokinase (PFK). Consistent with the upregulation of HK2 in KPCZ cells, total enzymatic HK activity was increased, whereas reduced expression of HK1 and HK2 in KPCmes cells was associated with decreased HK activity (Figure 1D, E, Suppl. Fig. 4A), in agreement with corresponding reductions in mRNA levels (Suppl. Fig. 4B). Unexpectedly, although most PFK family members (i.e., PFKM, PFKL and PFKP) were downregulated in KPCZ cells at both protein and mRNA levels (Suppl. Fig. 4C), PFK enzymatic activity was only moderately reduced as compared to KPCepi cells (Figure 1E). This partial preservation of activity was potentially compensated by concomitant upregulation of PKFB3 (Suppl. Fig. 4A and C) which positively regulates 6-phosphofructokinase 1 (PFK-1) activity [45].

Collectively, KPCZ cells do not show a defect until fructose-1,6-bisphosphate and maintain, or even moderately increase, steady-state glycolytic flux through elevated HK activity and increased expression of GLUT1 and PFKB3. However, further upregulation of glycolysis is not achievable, as reflected by the absence of glycolytic reserve, presumably because glycolytic enzymes already operate at near-maximal capacity—a hallmark of impaired metabolic plasticity.

### 3.2 Glucose tracing reveals impaired pyruvate kinase activity in low-colonizing cells

To gain deeper insight into the glycolytic alterations in KPCmes and KPCZ cells, we examined glycolytic kinetics using glucose tracing experiments with uniformly [U–^13^C]-labeled glucose (Figure 1F, Suppl. Fig. 4D). Cells were exposed to labeled glucose for 0.5, 1, 2, and 24 h, and incorporation into key metabolites was analyzed by mass spectrometry. Glucose uptake rates, as assessed by glucose m+6 labeling, were comparable across all cell lines (Suppl. Fig. 5A). Analysis of glycolytic intermediates revealed similar labeling kinetics up to phosphoenolpyruvate (PEP m+3) in all cell lines (Figure 1G, Suppl. Fig. 5A and B), indicating that KPCZ cells can compensate for reduced enzyme expression by increasing the activity of upstream glycolytic enzymes. In contrast, a marked reduction in labeled pyruvate (pyruvate m+3) was detected following 1 h of labeling in both low-colonizing cell types, KPCmes and KPCZ, suggesting diminished pyruvate kinase (PK) activity (Figure 1H, Suppl. Fig. 5C). These findings are consistent with reduced mRNA and protein levels of *Pklr*/PKLR in both cell types, decreased PKM protein abundance in KPCZ cells, as well as lower calculated PK activity based on the ratio of pyruvate m+3 to PEP m+3 (Figure 1H, Suppl. Fig. 5D). Reduced pyruvate availability resulted in a modest, yet not statistically significant decrease in lactate production in KPCmes cells, while lactate levels in KPCZ cells remained largely unchanged, in agreement with extracellular acidification rate (ECAR) measurements under steady-state glycolytic conditions (Suppl. Fig. 5E and F).

In summary, glycolytic flexibility is compromised in low-colonizing tumor cells, as reflected by distinct alterations in protein expression and enzymatic activity that converge on reduced pyruvate production. In particular, KPCZ cells exhibit compensatory upregulation of upstream glycolytic flux that cannot be further enhanced upon inhibition of oxidative phosphorylation, resulting in loss of glycolytic reserve and impaired metabolic plasticity.

### 3.3 TCA cycle and oxidative phosphorylation are altered in low-colonizing cells, resulting in distinct respiratory phenotypes in KPCZ and KPCmes cells

**Figure 2:**
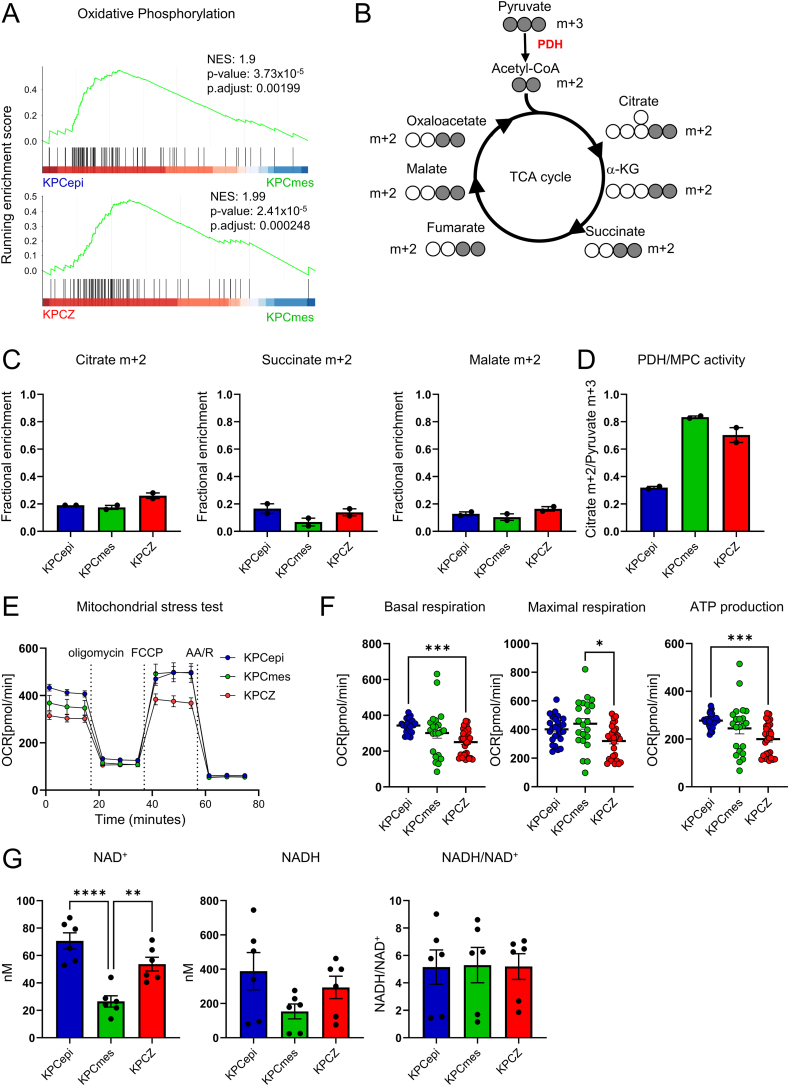
**Oxidative phosphorylation and NAD^+^ homeostasis are impaired in low-colonizing cells.** **A.** GSEA of the KEGG oxidative phosphorylation pathway from proteome analysis comparing KPCepi vs. KPCmes, and KPCZ vs. KPCmes cells reveals negative enrichment in KPCmes cells, with no significant enrichment observed in the KPCepi vs. KPCZ comparison. **B.** Schematic representation of [U–^13^C]-glucose labeling and tracing of resulting m+2 TCA cycle metabolites. Gray and open circles indicate number of [^13^C] and [^12^C] carbon atoms in each metabolite, respectively. **C.** Isotope tracing of TCA-related metabolites in KPCepi, KPCmes and KPCZ cell lines by mass spectrometry 1 h after addition of [U–^13^C]-glucose. **D.** Combined activity of pyruvate dehydrogenase (PDH) and mitochondrial pyruvate carrier (MPC) calculated as the ratio of citrate m+2/pyruvate m+3, showing increased activity in KPCmes and KPCZ. *n* = 2 biological replicates, 2 cell lines for each phenotype/genotype. **E.** and **F.** Mitochondrial stress test run in a Seahorse instrument measuring oxygen consumption rate (OCR) upon addition of oligomycin, carbonyl cyanide 4-(trifluoromethoxy)phenylhydrazone (FCCP) and antimycin A/rotenone (AA/R) (E) to calculate basal and maximal respiration and ATP production (F). *n* = 6 biological replicates, 2 cell lines for each phenotype/genotype, run in 5 technical replicates, showing that basal respiration, maximal respiration and ATP production is specifically reduced in KPCZ cells. **G.** Quantification of NAD^+^ and NADH concentrations and calculation of NADH/NAD^+^ ratios. *n* = 6 biological replicates, 2 cell lines for each phenotype/genotype. Data are represented as mean ± SEM. ∗*P* < 0.05, ∗∗*P* < 0.01, ∗∗∗*P* < 0.001, ∗∗∗∗*P* < 0.0001 by Kruskal–Wallis test (F) or one-way ANOVA with Tukey’s test (G).

KEGG pathway analysis of the proteomic data revealed a specific alteration of oxidative phosphorylation in KPCmes cells, whereas differences between KPCZ and KPCepi were comparatively modest (Figure 2A, Supplementary Tables 1 and 2). We therefore hypothesized that dysregulated glycolysis in low-colonizing cells might also impair oxidative phosphorylation and TCA cycle metabolism. To test this, we first analyzed the labeling kinetics of key TCA cycle metabolites following incubation with uniformly [U–^13^C]-labeled glucose (Figure 2B). Despite reduced PK activity and consequently less efficient pyruvate generation in KPCZ and KPCmes cells, the labeling kinetics of citrate m+2 remained unchanged as compared to KPCepi cells (Figure 2C, Suppl. Fig. 6A). Consistently, additional TCA cycle intermediates derived from pyruvate-dependent acetyl-CoA, including succinate m+2 and malate m+2, exhibited comparable labeling kinetics across all cell types (Figure 2C, Suppl. Fig. 6B and C). This observation suggests compensatory mechanisms. In fact, there was an upregulation of pyruvate dehydrogenase (PDH) and/or mitochondrial pyruvate carrier (MPC) activity in KPCZ and KPCmes cells to maintain mitochondrial acetyl-CoA supply (Figure 2D).

To functionally evaluate TCA cycle performance, and oxidative phosphorylation, we measured oxygen consumption rates (OCRs) using a mitochondrial stress test. Basal respiration and ATP-linked oxygen consumption were markedly reduced in KPCZ cells (Figure 2E, F, left), which confirmed previous observations [27]. Maximal respiratory capacity was also decreased in KPCZ cells, reaching statistical significance when compared to KPCmes cells (Figure 2E, F, middle). The cumulative reduction in respiratory parameters is in agreement with compromised mitochondrial respiratory capacity and ATP production in KPCZ cells (Figure 2E, F, right). Given the reduction in oxidative phosphorylation, we next assessed whether alterations in the mitochondrial NAD pool or redox balance could account for limited electron supply to the respiratory chain. While NAD^+^ and NADH levels were only moderately reduced in KPCZ cells, they were substantially decreased in KPCmes cells. Nevertheless, NADH/NAD^+^ ratios were only modestly altered or unchanged, indicating that mitochondrial electron transport remains largely effective in regenerating NAD^+^ in both cell types, despite overall reduced levels of NAD^+^ and NADH (Figure 2G).

In summary, low-colonizing KPCZ and KPCmes tumor cells maintain normal TCA cycle flux and NADH/NAD^+^ dynamics, consistent with preserved carbon flow through central metabolism via compensatory increases in PDH/MPC activity. However, the pronounced reduction in basal respiration and ATP production in KPCZ cells points to a specific dysregulation of mitochondrial respiratory function.

### 3.4 Mitochondria structure and function are impaired upon ZEB1 depletion

**Figure 3:**
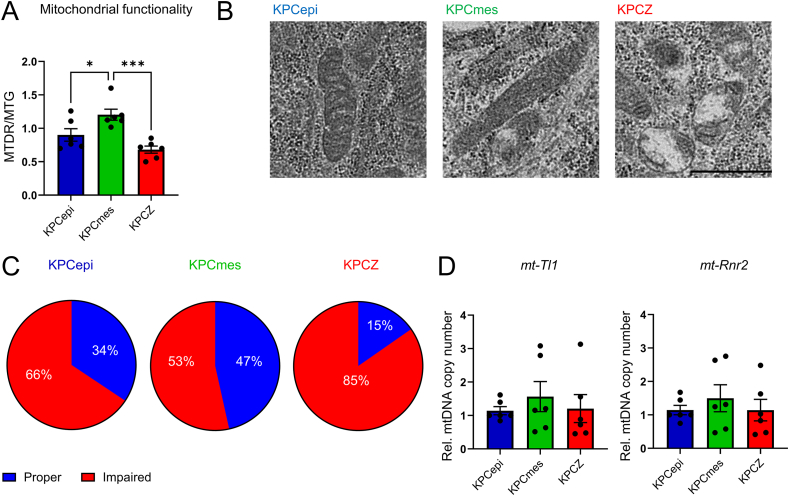
**ZEB1 deficiency disrupts mitochondrial morphology.** **A.** Ratio of mitochondrial membrane potential and mitochondrial biomass determined by MitoTracker deep red (MTDR) and MitoTracker green (MTG) mean fluorescence intensities, respectively, analyzed by flow cytometry, showing a reduction of mitochondrial membrane potential in KPCZ cells. *n* = 6 biological replicates, 2 cell lines for each phenotype/genotype. **B.** and **C.** Representative electron micrographs of KPC661, KPC550 and KPCZ346 cells at high magnification (B) and combined classification of mitochondria of KPCepi, KPCmes and KPCZ cell lines (C), showing overall impaired mitochondria structural integrity in KPCZ cells. Proper and impaired mitochondria morphologies were categorized as shown in Suppl. Fig. 7. **D.** Quantification of mitochondrial DNA copy numbers calculated by qPCR analysis of measuring mitochondrially encoded DNA copies for tRNA leucine 1 (UUA/G) (*mt-Tl1*) and 16S RRNA (*mt-Rnr2*) normalized to nuclear DNA content by *Gapdh*. *n* = 6 biological replicates, 2 cell lines for each phenotype/genotype. Scale bar, 1 μm. Data are represented as mean ± SEM. ∗*P* < 0.05, ∗∗∗*P* < 0.001, by one-way ANOVA with Tukey’s (A) test. (For interpretation of the references to color in this figure legend, the reader is referred to the Web version of this article.)

To determine whether the reduced oxidative phosphorylation observed in KPCZ cells results from mitochondrial defects, we first assessed mitochondrial biomass and membrane potential by flow cytometry using MitoTracker green (MTG) and MitoTracker deep red (MTDR). The ratio of mitochondrial membrane potential to biomass (MTDR/MTG) was highest in KPCmes cells (Figure 3A), indicating a higher proportion of functionally active mitochondria. In contrast, KPCZ cells exhibited the lowest membrane potential–to–biomass ratio, suggesting a lower proportion of functionally active mitochondria following *Zeb1* deletion. To substantiate these findings, we performed ultrastructural analyses of mitochondria in all cell lines based on standardized morphological criteria [41] (Suppl. Fig. 7A). KPCmes displayed the most organized mitochondria architecture reaching almost 50% of mitochondria classified as ‘morphologically intact’ in agreement with previous data from cancer cells [46] (Figure 3B, C, Suppl. Fig. 7B–D). KPCepi cells showed a modest reduction, with approximately 35% of mitochondria exhibiting normal structure (Figure 3B, C, Suppl. Fig. 7B–D). In striking contrast, KPCZ cells demonstrated a pronounced structural imbalance, with only ∼15% of mitochondria appearing morphologically intact (Figure 3B, C, Suppl. Fig. 7B–D). The majority of mitochondria in KPCZ cells were characterized by irregular, rounded morphologies, swollen cristae, and loss of intact cristae junctions, all hallmarks of mitochondrial dysfunction (Figure 3B, Suppl. Fig. 7B–D). Importantly, quantification of extrachromosomal mitochondrial DNA, used as a proxy for mitochondrial abundance, revealed comparable mitochondrial content across all cell lines, with a trend toward increased mitochondrial numbers in KPCmes cells (Figure 3D).

These findings indicate that mitochondrial biogenesis and overall mitochondrial mass are largely preserved. However, mitochondrial homeostasis and cristae organization are profoundly disrupted in KPCZ cells, leading to compromised mitochondrial structure and function.

### 3.5 NADPH generation is downregulated in low-colonizing cells

**Figure 4:**
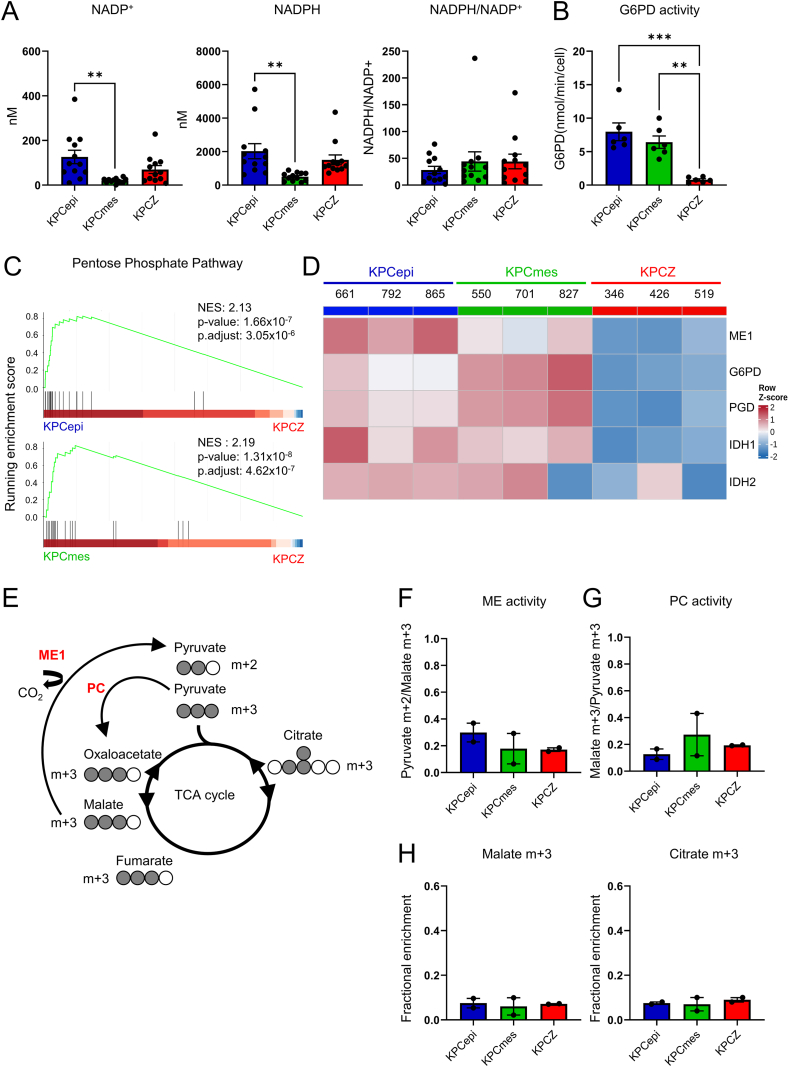
**NADPH regeneration and anaplerotic enzyme activities are limited in low colonizing tumor cells.** **A.** Quantification of NADP^+^ and NADPH and calculation of relative NADPH/NADP^+^ ratios in KPCepi, KPCmes and KPCZ cells, showing an overall reduction of nicotinamide adenine dinucleotide phosphate in KPCZ cells and even lower levels in KPCmes cells. *n* = 12 biological replicates, 2 cell lines for each phenotype/genotype. **B.** Quantitative analysis of the enzymatic activity of glucose-6-phosphate-dehydrogenase (G6PD) as indicator of pentose phosphate pathway activity, showing specific impairment in KPCZ cells. *n* = 6 biological replicates, 2 cell lines for each phenotype/genotype. **C.** GSEA of the KEGG pentose phosphate pathway from proteome analysis comparing KPCepi vs. KPCZ and KPCmes vs. KPCZ cells indicating negative enrichment in KPCZ cells. **D.** Heatmap of protein expression of cell lines as indicated showing relative abundance of key enzymes involved in NADPH generation quantified by mass spectrometry and visualized as row z-score–scaled values. **E.** Schematic representation of [U–^13^C]-glucose labeling and tracing of resulting m+3 TCA cycle metabolites that are generated by pyruvate carboxylase (PC) activity, while malic enzyme 1 generates m+2 pyruvate. Gray and open circles indicate number of [^13^C] and [^12^C] carbon atoms in each metabolite, respectively. **F.** and **G.** Relative activity of malic enzyme (ME) (F) and pyruvate carboxylase (PC) (G) calculated by ratios of pyruvate m+2 and malate m+3 and of malate m+3 and pyruvate m+3, respectively, measured by isotype tracing 1 h after addition of [U–^13^C]-glucose by mass spectrometry. While ME activity shows a moderate decrease in KPCZ and KPCmes cells, PC activity is slightly increased in KPCZ and KPCmes cells. *n* = 2 biological replicates, 2 cell lines for each phenotype/genotype. **H.** Isotope tracing of m+3 TCA-related metabolites 1 h after addition of [U–^13^C]-glucose measured by mass spectrometry reveals equal amounts of malate m+3 and citrate m+3 in all cell lines. *n* = 2 biological replicates, 2 cell lines for phenotype/genotype. Data are represented as mean ± SEM. ∗*P* < 0.05, ∗∗*P* < 0.01, ∗∗∗*P* < 0.001 by one-way ANOVA with Tukey’s test (A and B).

In addition to fueling the electron transport chain, TCA cycle intermediates provide essential substrates for NADPH production, which is required to maintain redox balance and an effective ROS scavenging system [47,48]. Because efficient lung colonization depends critically on reductive biosynthesis and resistance to oxidative stress [13,14,49,50] we investigated whether mitochondrial dysfunction and reduced ATP production compromise NADPH generation and redox homeostasis in low-colonizing cells. Absolute levels of both NADP^+^ and NADPH were significantly reduced in KPCmes cells, with a similar trend observed in KPCZ cells. However, NADPH/NADP^+^ ratios were comparable across all cell types, with a tendency toward the lowest ratios in KPCepi cells, indicating that neither impaired NADPH recycling nor excessive consumption occurred in KPCmes and KPCZ cells under steady-state conditions (Figure 4A). Given the reduced total NADPH levels in both low-colonizing cell lines, we next examined the pathways responsible for NADPH production. NADPH is primarily generated from glucose via the oxidative pentose-phosphate pathway (PPP) with additional contributions from malic enzyme (ME) [51,52] and cytosolic and mitochondrial isocitrate dehydrogenase (IDH) [[53], [54], [55]] (Suppl. Fig. 8A). Activity of the rate-limiting PPP enzyme glucose-6-phosphate dehydrogenase (G6PD) was significantly decreased in KPCZ, but remained unchanged in KPCmes cells (Figure 4B). KEGG pathway analysis revealed a significant downregulation of the PPP in KPCZ cells compared with both KPCepi and KPCmes cells (Figure 4C, Supplementary Tables 1 and 2). In line with the diminished enzyme activity, we observed reduced protein levels of G6PD and phosphogluconate dehydrogenase (PGD) in KPCZ cells (Figure 4D, Suppl. Fig. 8B, Supplementary Table 1). In addition, protein levels of ME1, IDH1 and IDH2 were reduced in KPCZ as well as in KPCmes cells, further limiting NADPH production in both the cytosol and mitochondria (Figure 4D, Suppl. Fig. 8B and C, Supplementary Table 1).

To assess ME activity, we analyzed [U–^13^C]-glucose tracing data, focusing on m+3–labeled TCA cycle intermediates generated via pyruvate carboxylase (PC) activity and subsequently converted by ME to pyruvate m+2 (Figure 4E). ME activity was calculated from the ratio of pyruvate m+2 to malate m+3 while PC activity was inferred from the ratio of malate m+3 to pyruvate m+3. We found a moderate decrease in ME activity in line with lower mRNA and protein expression of ME1 in KPCZ and KPCmes cells (Figure 4D, F, Suppl. Fig. 8C). Notably, PC activity was slightly increased in the low-colonizing cells (Figure 4G), whereas malate m+3 and citrate m+3 levels remained unchanged (Figure 4H, Suppl. Fig. 8D), overall suggesting that NADPH generation via ME activity may be moderately decreased.

In summary, KPCZ cells preferentially channel glucose toward glycolysis, while PPP and ME activities are insufficient to sustain efficient NADPH production. Although PPP activity remains comparatively preserved in KPCmes cells, NADPH levels do not reach those observed in KPCepi cells. While the overall reduction in NADPH abundance in KPCZ and KPCmes cells does not cause a pronounced imbalance in NADPH/NADP^+^ ratios under steady-state conditions, it is likely to render these cells particularly vulnerable during metabolic stress, such as that encountered during lung colonization, when oxygen levels and NADPH demands are high.

### 3.6 Specific metabolic genes are directly regulated by ZEB1

To gain insight into how ZEB1 controls metabolic plasticity at the level of gene expression, we first analyzed RNA-Seq data from TGFβ-treated KPC792 and KPCZ346 cells. A 6-d treatment of KPCepi cells resulted in a strong decrease in expression of metabolic genes, while KPCZ cells did not respond to TGFβ (Suppl. Fig. 9A) [27], indicating an immediate change in gene expression mediated by ZEB1 increase and EMT induction. We checked binding of ZEB1 to the corresponding loci using published ZEB1 ChIP-seq data sets of human cancer cell lines [[33], [34], [35], [36], [37], [38]] and found ZEB1 peaks at *PKLR*, *IDH1* and *ME1* loci of various cell lines (Suppl. Fig. 9B). To confirm direct regulation by ZEB1 we analyzed immediate effects on gene expression in KPCepi cell lines by qRT-PCR following a 48-h knockdown of *Zeb1*. In both cell lines *Pklr*, *Me1* and *Idh1* mRNA levels were decreased, while *G6pdx* remained largely unaffected, indicating a direct regulation of these metabolic genes except for *G6pdx* (Suppl. Fig. 9C). These data indicate that ZEB1 is regulating gene expression of specific metabolic genes, like *Pklr* and *Me1* directly, whereas others appear to be subject to more indirect regulation in course of ZEB1-mediated reprogramming during EMT, underlining the complexity of these processes.

### 3.7 Capacity to cope with redox stress is impaired in low-colonizing cells

**Figure 5:**
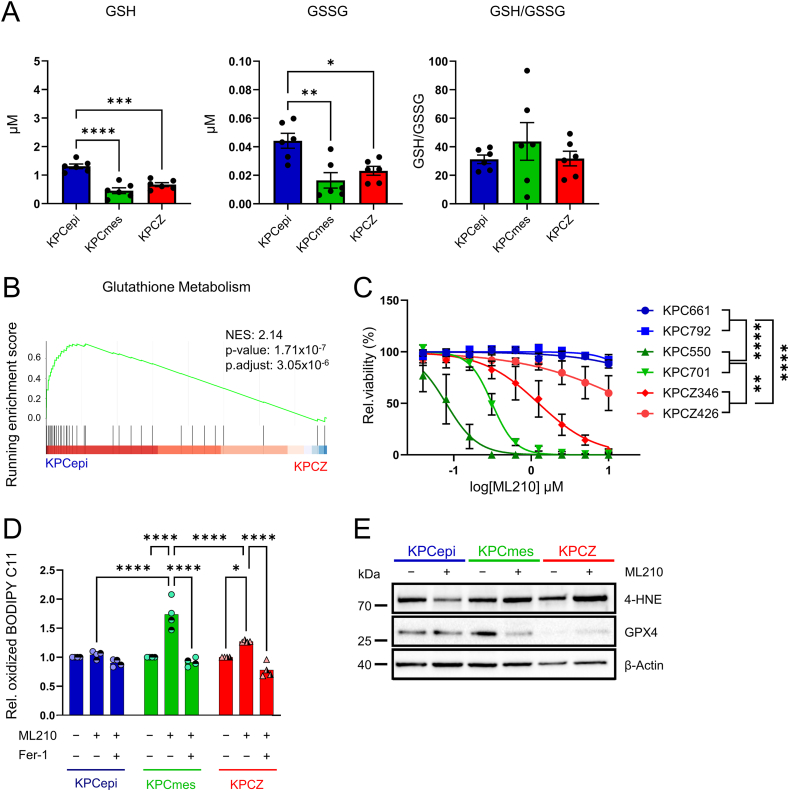
**ZEB1 differentially regulates redox pathways to sustain glutathione, thereby conferring increased ferroptosis sensitivity in low-colonizing cells.** **A.** Quantification of GSH and GSSG concentrations and calculation of GSH/GSSG ratios, demonstrating that low colonizing cells have overall reduced glutathione levels, but ratios are maintained under steady-state conditions. *n* = 6 biological replicates, 2 cell lines for each phenotype/genotype. **B.** GSEA of the KEGG glutathione metabolism pathway from proteome analysis comparing. KPCepi vs. KPCZ cells showing negative enrichment in KPCZ cells. **C.** Relative viability of KPCepi, KPCmes and KPCZ cells treated with increasing concentrations of ML210, showing that KPCmes cells are very vulnerable and KPCepi cells are very resistant to GPX4 inhibition, while KPCZ cells show sensitivities in between. *n* = 4 biological replicates, 2 cell lines for each phenotype/genotype. Data are represented as relative confluence after 69 h, measured by live-cell imaging with an Incucyte S3. Data are represented as mean ± SEM. ∗*P* < 0.05, ∗∗*P* < 0.01, ∗∗∗*P* < 0.001, ∗∗∗∗*P* < 0.0001 by one-way ANOVA with Tukey’s test (A) and by two-way analysis of variance (ANOVA) (C). **D.** Relative oxidized BODIPY 581/591C11 of KPCepi, KPCmes and KPCZ cells treated with ML210, showing that KPCmes and to a lesser extend also KPCZ cells have increased lipid peroxidation which is rescued by Ferrostatin-1. *n* = 4 biological replicates, 2 cell lines for each phenotype/genotype. Filled and half-filled symbols indicate treatment with 1 μM ML210 for 1 h and 0.2 μM ML210 for 2 h, respectively. Data are represented as mean ± SEM. ∗*P* < 0.05, ∗∗∗∗*P* < 0.0001 by two-way ANOVA with Tukey’s test. **E.** Representative immunoblots of ferroptosis markers in KPCepi, KPCmes and KPCZ cells after treatment with 1 μM ML210 for 4 h. *n* = 1 biological replicate.

NADPH is an essential cofactor for maintaining the reduced pool of cellular ROS scavengers, particularly the glutathione (GSH) pool through the glutathione reductase reaction, and alterations in NADPH availability are therefore expected to directly affect cellular redox balance and the capacity to detoxify ROS [56,57]. To determine whether the reduced NADPH levels observed in KPCZ and KPCmes cell lines result in redox imbalance, we quantified the intracellular levels of reduced (GSH) and oxidized (GSSG) glutathione. Both GSH and GSSG levels were markedly decreased in the low-colonizing cells despite a trend toward increased GSH/GSSG ratios (Figure 5A). Consistent with this observation, KEGG pathway analysis of the proteomic data identified glutathione metabolism as specifically altered in KPCZ cells (Figure 5B, Supplementary Tables 1 and 2). These findings suggest that, although steady-state redox ratios appear largely preserved, the overall depletion of the glutathione pool compromises ROS-scavenging capacity in KPCZ and KPCmes cells.

To functionally test this hypothesis, we mimicked increased ROS-mediated stress encountered during lung colonization conditions – known to trigger ferroptotic cell death particularly in mesenchymal tumor cells [42,58,59] – by treating cells with the GPX4 inhibitor and ferroptosis inducer ML210. In line with previous reports, efficiently colonizing KPCepi cells remained viable even at high ML210 concentrations, consistent with their resistance to ferroptosis. In contrast, KPCmes cells displayed pronounced sensitivity to ferroptotic cell death (Figure 5C) [42]. Notably, KPCZ cells also exhibited increased susceptibility to ferroptosis despite maintaining an overall epithelial morphology similar to KPCepi cells. This was in line with changes in ferroptosis-related markers, like increased lipid peroxidation and 4-HNE in KPCmes, but also in KPCZ cells, combined with a reduction of GPX4 in KPCmes under ML210 treatment and an overall lower basal level of GPX4 in KPCZ cells (Figure 5D, E). This heightened ferroptosis sensitivity indicates that impaired antioxidant capacity in KPCZ cells limits their ability to survive elevated ROS stress, such as that encountered during lung colonization.

In conclusion, ZEB1-dependent rewiring of antioxidant and redox-regulatory pathways generates distinct redox states that correlate with metastatic colonization efficiency. The ability to maintain redox homeostasis under conditions of metabolic and oxidative stress thus emerges as a critical determinant of successful lung colonization.

## 4 Discussion

Efficient metastatic colonization of distant organs by disseminated tumor cells requires a high degree of cellular plasticity and metabolic adaptability to withstand hostile microenvironmental conditions. In particular, successful metastatic outgrowth depends on the ability of tumor cells to maintain redox homeostasis and prevent excessive accumulation of reactive oxygen species, thereby avoiding oxidative stress-induced cell death, including ferroptosis [[60], [61], [62], [63]]. Here we investigated, how the metabolism differs among pancreatic cancer cell lines derived from the KPC mouse model with distinct metastatic capacities and degrees of cellular plasticity. Specifically, we compared highly efficient lung colonizers with pronounced plasticity (KPCepi) to poorly colonizing cell lines that either lack the key regulator of EMT, cellular plasticity, and metastasis, ZEB1 (KPCZ), or exhibit a highly undifferentiated mesenchymal phenotype (KPCmes). We show that depletion of ZEB1 leads to profound loss of glycolytic capacity, thereby preventing a compensatory switch to glycolysis upon inhibition of oxidative phosphorylation. This defect is accompanied by mitochondrial abnormalities that impair ATP production without substantially affecting TCA cycle metabolite flux. In contrast, although low-colonizing KPCmes cells exhibit reduced basal glycolysis, they retain normal glycolytic reserve and display intact mitochondria function with preserved oxidative phosphorylation. Strikingly, both KPCZ and KPCmes cells show reduced levels of NADPH and GSH, compromising their ability to efficiently neutralize ROS under stress conditions. Accordingly, both cell lines are more vulnerable to inhibition of GPX4 and show increased susceptibility to ferroptotic cell death. Together, these findings indicate that metastatic competence requires the coordinated activity and adaptability of distinct metabolic pathways. Disruption of these pathways compromises metabolic and cellular plasticity, limits redox buffering capacity, and ultimately reduces the ability of tumor cells to withstand oxidative stress.

The capacity to adapt cellular metabolism—through dynamic regulation of glycolysis and oxidative phosphorylation, utilization of diverse nutrient sources, and maintenance of redox homeostasis—has been linked to unlimited proliferative potential and efficient metastatic dissemination [64,65]. Numerous metabolic pathways that enable cancer cells to adapt to the nutrient landscapes of specific metastatic niches have been described [66], including utilization of extracellular pyruvate to promote lung colonization in breast cancer [67]. However, the molecular requirements that confer general metabolic plasticity enabling tumor cells to survive microenvironmental challenges during metastasis remain incompletely understood. Here, we identified a coordinated requirement for flexible glycolytic and oxidative metabolism, intact mitochondrial homeostasis, and robust antioxidant capacity as key determinants of efficient lung colonization.

Despite preserved TCA cycle metabolites in KPCZ cells, supported by increased PDH and MPC activity, ATP production is compromised due to mitochondrial dysfunction and damage. The importance of functional mitochondria for lung colonization has been demonstrated previously [68,69], yet the mechanisms underlying mitochondrial defects in KPCZ cells remain unclear. Our data suggest impaired regulation of mitochondrial dynamics, including fission, fusion, and mitophagy. Notably, protein levels of MFN2 as one important fusion regulator are increased in KPCZ cells (Supplementary Table 1), consistent with prior evidence linking ZEB1 to *Mfn2* regulation [[70], [71], [72]].

In addition, KPCZ cells exhibit markedly reduced expression and activity of G6PD, the rate-limiting enzyme of the PPP and a major source of NADPH required for oxidative stress resistance. Previous work showed that during metastasis of melanoma cells the activity of the PPP enzyme G6PD is crucial to cope with increased oxidative stress and the block of G6PD leads to elevation of ME1 activity [28]. Combined with low activity of alternative NADPH-generating enzymes and diminished glutathione levels, this defect likely underlies the reduced metastatic capacity of KPCZ cells. These cells further display downregulation of additional antioxidant systems, including thioredoxins and peroxiredoxins (Supplementary Table 1) when compared to KPCepi, exacerbating redox vulnerability [73,74].

In contrast to KPCZ cells, KPCmes retain intact mitochondrial function and PPP activity but exhibit reduced basal glycolysis and profoundly diminished NADPH and glutathione pools. Since escaping ferroptotic cell death during lung colonization is crucial [[61], [62], [63]], it is likely that both KPCmes and KPCZ cells succumb to ferroptotic cell death, albeit through distinct mechanisms. It was shown that in mesenchymal-like cells such as KPCmes, heightened sensitivity to ferroptosis is driven by altered membrane lipid composition enriched in polyunsaturated phospholipids (PUFAs) [42,75]. As demonstrated by manipulation of lipogenic enzymes, high PUFA ratios renders KPCmes highly dependent on GPX4-mediated detoxification to prevent lethal accumulation of lipid-derived reactive oxygen species [42,76]. Notably, in comparison to KPCepi cells and in spite of their more epithelial phenotype, KPCZ cells exhibit a moderate increase in PUFAs [42], which together with their impaired antioxidant capacity may accelerate lipid peroxidation and ferroptotic cell death.

Collectively, our findings indicate that insufficient NADPH and glutathione availability compromise redox homeostasis and limits lung colonization efficiency. Challenging redox balance through GPX4 inhibition confirmed heightened ferroptosis sensitivity in both KPCmes and KPCZ cells, underscoring the requirement for balanced antioxidant systems in metastatic competence. Future studies will be required to determine whether targeting metabolic plasticity and redox buffering represents a viable therapeutic strategy to prevent lung metastasis in pancreatic ductal adenocarcinoma and other cancer types.

## CRediT authorship contribution statement

**Ece Grace:** Writing – review & editing, Writing – original draft, Visualization, Validation, Methodology, Investigation. **Deyu Zou:** Visualization, Validation, Methodology, Investigation. **Romy Böttcher-Loschinski:** Writing – review & editing, Visualization, Validation, Methodology, Investigation. **Martin Böttcher:** Writing – review & editing, Visualization, Validation, Methodology, Investigation. **Harald Schuhwerk:** Writing – review & editing, Validation, Methodology, Funding acquisition. **Yussuf Hajjaj:** Visualization, Validation, Methodology, Investigation, Data curation. **Annemarie Schwab:** Validation, Methodology, Investigation. **Simon Brandt:** Validation, Methodology, Investigation. **Ana Clavel Ezquerra:** Validation, Methodology. **Witold Szymanski:** Writing – review & editing, Visualization, Validation, Methodology, Investigation, Data curation. **Johannes Graumann:** Validation, Methodology, Data curation. **Philipp Arnold:** Writing – review & editing, Visualization, Validation, Methodology, Investigation. **Renato Liguori:** Writing – review & editing, Validation, Methodology, Data curation. **Fulvia Ferrazzi:** Writing – review & editing, Validation, Methodology, Data curation. **Constantin P. Krempe:** Visualization, Validation, Methodology, Data curation. **Luiza Martins Nascentes Melo:** Writing – review & editing, Validation, Methodology, Data curation. **Gabriele Allies:** Validation, Methodology. **Sven W. Meckelmann:** Validation, Methodology. **Dirk Mielenz:** Writing – review & editing, Validation, Methodology. **Simone Brabletz:** Writing – review & editing, Validation, Methodology, Funding acquisition. **Dimitrios Mougiakakos:** Validation, Supervision, Methodology, Funding acquisition, Conceptualization. **Alpaslan Tasdogan:** Writing – review & editing, Writing – original draft, Visualization, Validation, Supervision, Methodology, Data curation, Conceptualization. **Thomas Brabletz:** Writing – review & editing, Writing – original draft, Visualization, Validation, Supervision, Methodology, Funding acquisition, Conceptualization. **Marc P. Stemmler:** Writing – review & editing, Writing – original draft, Visualization, Validation, Supervision, Methodology, Funding acquisition, Conceptualization.

## Funding sources

This work was supported by the 10.13039/501100001659German Research Foundation (TRR417-Project ID: 540805631-TP04, TRR305-Project ID: 429280966-TP A03, A04, B01, B07 and Z01; 290599057, 314375996, 461704629 (SPP2306), 511711508, 416775465, and 555537070), the 10.13039/100008672Wilhelm Sander-Stiftung (2020.039.1) and IZKF-Erlangen (IZKF P34, P133 and D39). A.T. was supported by an Emmy Noether Award from the 10.13039/501100001659German Research Foundation (DFG, 467788900) and the Ministry of Culture and Science of the 10.13039/100030848State of North Rhine-Westphalia (NRW-Nachwuchsgruppenprogramm). A.T. acknowledges the support of an ERC starting grant (grant no. METATARGET, 101078355). A.T. holds the Peter Hans Hofschneider of Molecular Medicine endowed professorship by the Stiftung Experimentelle Biomedizin.

## Declaration of competing interest

Authors declare that they have no competing interests.

## Figures and Tables

**Figure:**
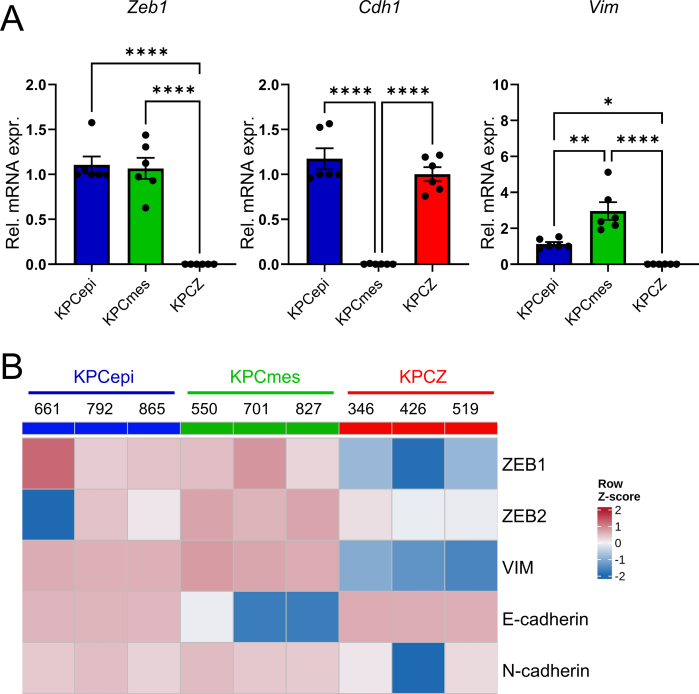
**Supplementary Figure 1: KPC cells can be discriminated into epithelial and mesenchymal phenotypes.** **A.** Relative mRNA expression levels of *Zeb1*, E-cadherin (*Cdh1)* and Vimentin (*Vim*) analyzed by qRT-PCR, demonstrating KPCepi and KPCmes features and absence of *Zeb1* and increase in *Cdh1/Vim* ratios in KPCZ cells. *n* = 6 biological replicates, 2 cell lines for each phenotype/genotype. Expression was normalized to *Actb* and represented relative to KPC661. **B.** Heatmap of protein expression of cell lines as indicated showing relative abundance of selected markers for epithelial–mesenchymal transition (EMT) quantified by mass spectrometry and visualized as row z-score–scaled values. Data are represented as mean ± SEM. ∗*P* < 0.05, ∗∗*P* < 0.01, ∗∗∗∗*P* < 0.0001 by one-way ANOVA with Tukey’s test (A).

**Figure:**
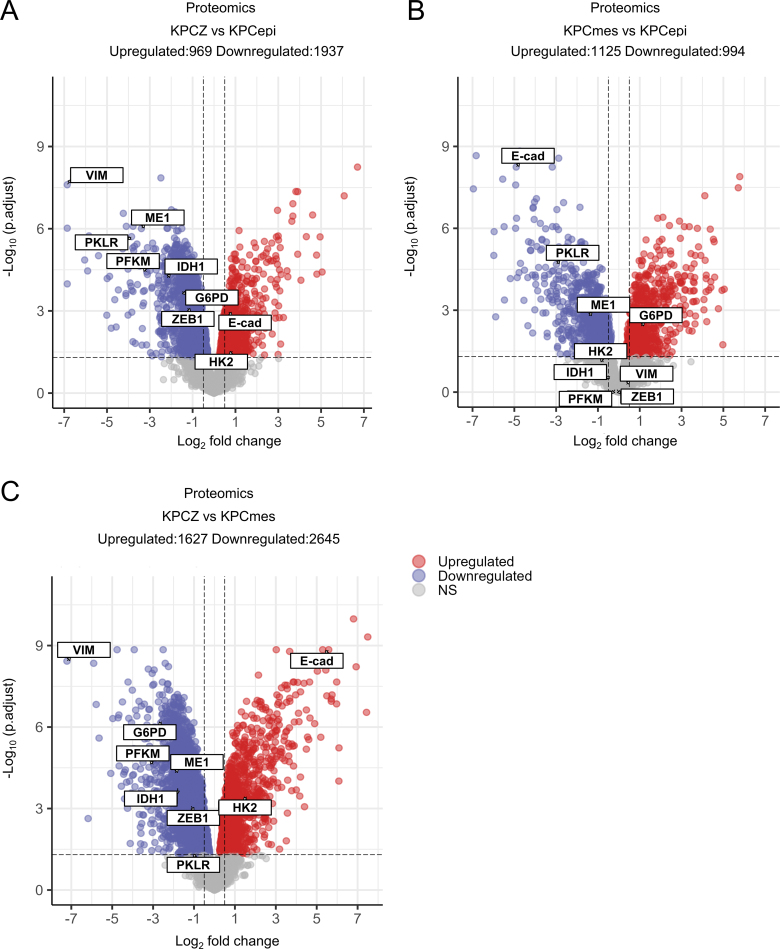
**Supplementary Figure 2: Volcano plot of proteome data of KPCepi, KPCmes and KPCZ cells.** **A-C.** Volcano plots showing significantly up- and downregulated proteins comparing KPCZ vs. KPCepi (A) KPCmes vs. KPCepi (B) and KPCZ vs. KPCmes (C). *n* = 9 biological replicates, 3 cell lines for each phenotype/genotype. Total number of identified proteins = 8026.

**Figure:**
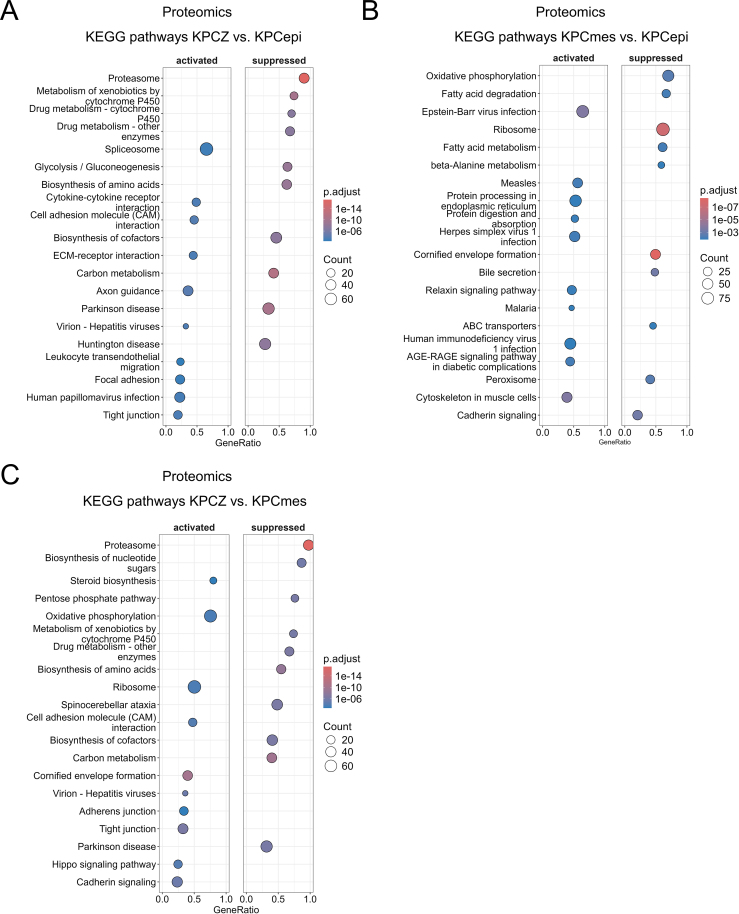
**Supplementary Figure 3: KEGG pathway analysis of KPCepi, KPCmes and KPCZ cells.** **A-C.** Dot plots showing the top 20 enriched KEGG pathways based on mass spectrometry quantification of protein expression, comparing KPCZ vs. KPCepi (A), KPCmes vs. KPCepi (B) and KPCZ vs. KPCmes (C). Dot size represents the number of proteins contributing to each pathway, and color indicates the adjusted *P*-value. *n* = 9 biological replicates, 3 cell lines for each phenotype/genotype.

**Figure:**
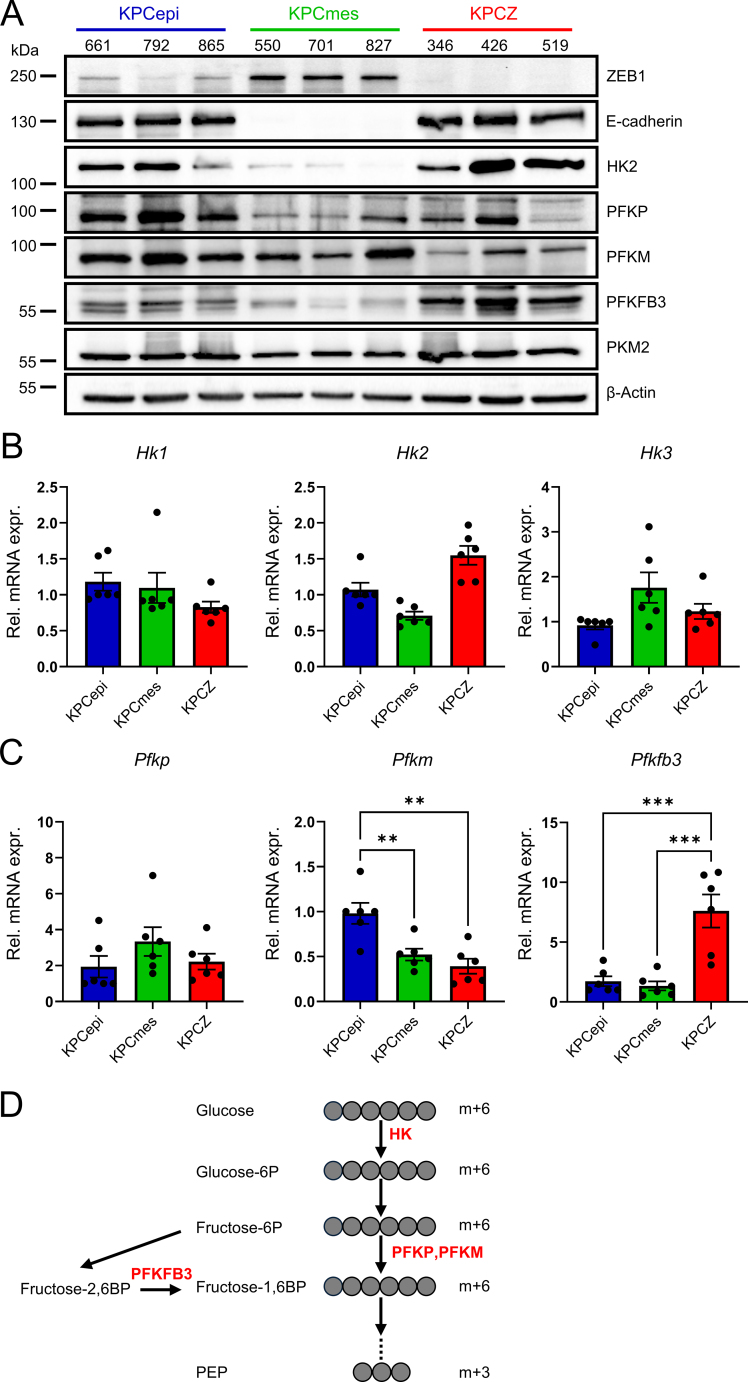
**Supplementary Figure 4: Protein and mRNA expression of genes involved in glycolysis is altered in low colonizing cells.** **A.** Representative immunoblots of glycolytic enzymes in KPCepi, KPCmes and KPCZ cells. **B.** Relative mRNA expression levels of hexokinase 1 (*Hk1),* hexokinase 2 (*Hk2)* and hexokinase 3 (*Hk3)* analyzed by qRT-PCR. **C.** Relative mRNA expression levels of phosphofructokinase platelet (*Pfkp*), phosphofructokinase muscle (*Pfkm*) and 6-phosphofructo-2-kinase/fructose-2,6-biphosphatase 3 (*Pfkfb3*) analyzed by qRT-PCR. *n* = 6 biological replicates, 2 cell lines for each phenotype/genotype. Expression was normalized to *Actb* and represented relative to KPC661. **D.** Schematic representation of glycolysis-related metabolites. Data are represented as mean ± SEM. ∗*P* < 0.05, ∗∗*P* < 0.01, ∗∗∗*P* < 0.001, ∗∗∗∗*P* < 0.0001 by one-way ANOVA with Tukey’s test (B to C).

**Figure:**
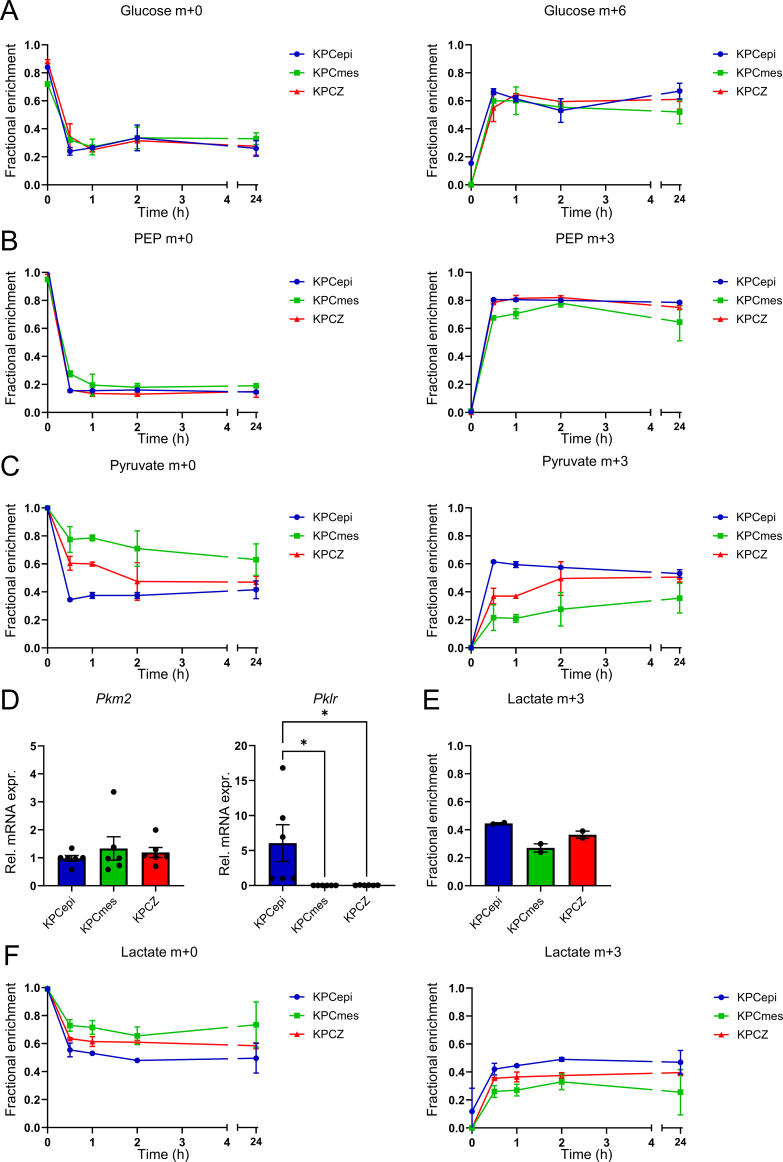
**Supplementary Figure 5: [U-^13^C]-glucose tracing kinetics of glycolysis-related metabolites.** **A**-**C.** Isotope tracing of unlabeled and labeled fractions of glycolysis-related metabolites 0.5, 1, 2 and 24 h after addition of [U-^13^C]-glucose measured by mass spectrometry in KPCepi, KPCmes and KPCZ cell lines, represented as fractional enrichment for glucose (A), PEP (B) and pyruvate (C). 2 cell lines for each phenotype/genotype. **D.** Relative mRNA expression levels of pyruvate kinase m2 (*Pkm2)* and pyruvate kinase liver and red blood cell (*Pklr*) analyzed by qRT-PCR. *n* = 6 biological replicates, 2 cell lines for each phenotype/genotype. Expression was normalized to *Actb* and represented relative to KPC661. **E.** Isotope tracing of lactate m+3 in KPCepi, KPCmes and KPCZ cell lines by mass spectrometry analysis 1 hour after addition of [U-^13^C]-glucose. **F.** Isotope tracing of unlabeled (m+0) and labeled lactate (m+3) 0.5, 1, 2 and 24 h after addition of [U-^13^C]-glucose measured by mass spectrometry in KPCepi, KPCmes and KPCZ cell lines, represented as fractional enrichment. 2 cell lines for each phenotype/genotype. Data are represented as mean ± SEM. ∗*P* < 0.05 by one-way ANOVA with Tukey’s test (D).

**Figure:**
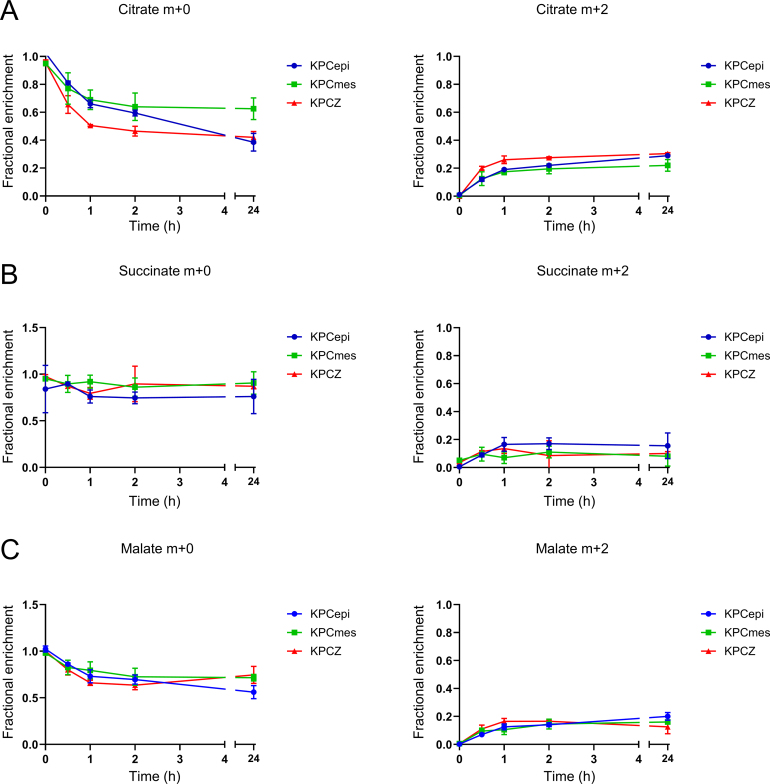
**Supplementary Figure 6: [U–^13^C]-glucose tracing kinetics of TCA-related metabolites.** **A**-**C.** Isotope tracing of unlabeled and m+2 labeled TCA-related metabolites 0.5, 1, 2 and 24 h after addition of [U–^13^C]-glucose measured by mass spectrometry in KPCepi, KPCmes and KPCZ cell lines, represented as fractional enrichment for citrate (A), succinate (B) and malate (C). 2 cell lines for each phenotype/genotype.

**Figure:**
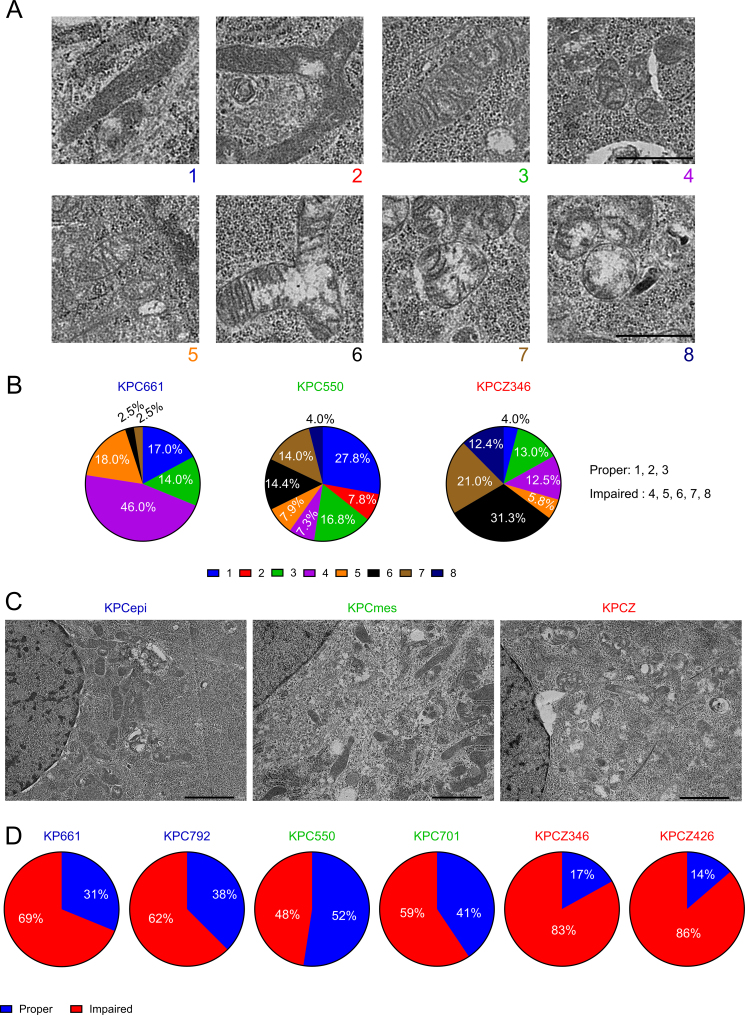
**Supplementary Figure 7: Analysis of mitochondria morphology reveals increased populations of impaired mitochondria in KPCZ cells.** **A.** Representative micrographs showing examples of subclassification of mitochondria found in any of the different cell lines. The subcategories were determined according to cristae structure (intact) and cristae number per mitochondria in electron micrographs according to previous analysis leading to categories 1, 2 and 3 as proper and categories 4, 5, 6, 7 and 8 as impaired mitochondria [41]. See methods for details. **B.** KPC661, KPC550 and KPCZ346 cells analyzed for the proportion of each classification represented as pie charts. *n* = 3 biological replicates. **C.** Representative electron micrographs at low magnification of KPC661, KPC550 and KPCZ346 cell lines. Scale bar, 2 μm. **D.** Distribution of impaired and proper mitochondria for KPCepi, KPCmes and KPCZ cell lines based on the classification in (B), showing overall impaired mitochondria structural integrity in KPCZ cells.

**Figure:**
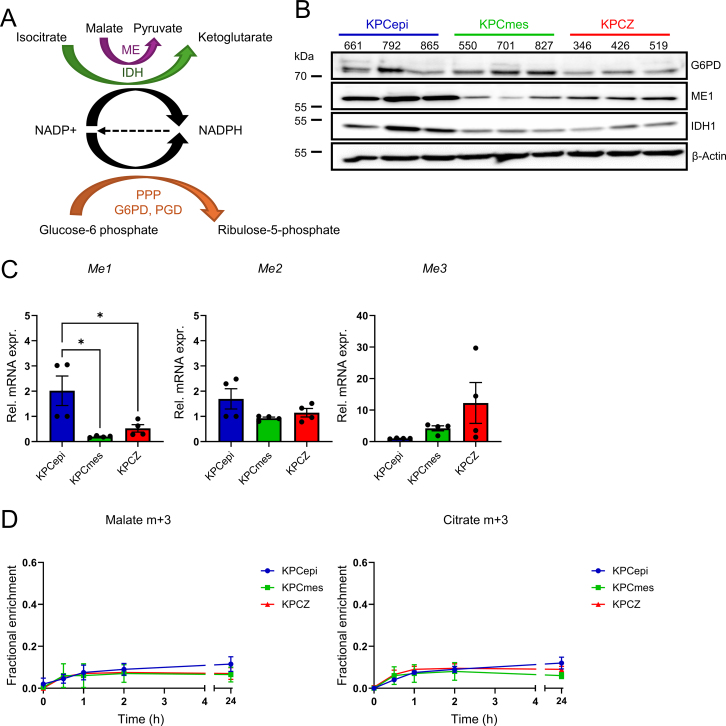
**Supplementary Figure 8: Canonical NADPH production pathways are differentially regulated in low colonizing cells.** **A.** Schematic representation of the main canonical NADPH production pathways ME, IDH and PPP (adapted from [54]). **B.** Representative immunoblots of enzymes involved in NADPH production in KPCepi, KPCmes and KPCZ cells. **C.** Relative mRNA expression levels of malic enzyme 1 (*Me1*), malic enzyme 2 (*Me2*) and malic enzyme 3 (*Me3*). for *n* = 4 biological replicates, 2 cell lines for each phenotype/genotype. Expression was normalized to *Actb* and represented relative to KPC661. **D.** Isotope tracing of m+3 labeled TCA-related metabolites 0.5, 1, 2 and 24 h after addition of [U–^13^C]-glucose measured by mass spectrometry in KPCepi, KPCmes and KPCZ cell lines, represented as fractional enrichment. 2 cell lines for each phenotype/genotype. Data are represented as mean ± SEM. ∗*P* < 0.05 by one-way ANOVA with Tukey’s test (B).

**Figure:**
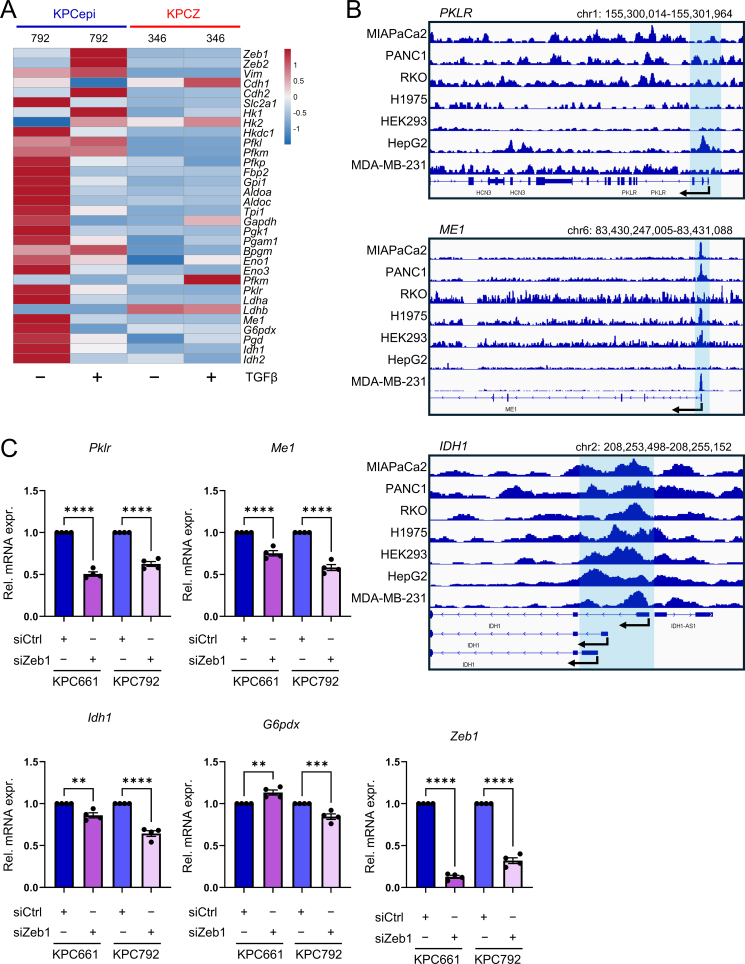
**Supplementary Figure 9: ZEB1 directly regulates specific metabolic genes.** **A.** RNA-seq analysis of KPCepi (KPC792) and KPCZ (KPCZ346) cells treated with 5 ng/ml TGFβ for 6 days. Heatmap showing downregulation of many key enzymes involved in glycolysis and NAPDH generation exclusively in KPCepi, while EMT genes are upregulated. Note, that gene expression in KPCZ cells is almost unaffected. Values are given as row z-scores. **B.** Binding of ZEB1 to selected gene loci of metabolic enzymes *PKLR*, *ME1* and *IDH1*, visualized from publicly available ZEB1 ChIP-seq datasets of human (cancer) cell lines as indicated. Highlighted regions indicate robust ZEB1 binding in the majority of analyzed cell lines. Transcription start sites are indicated by vertical arrows. **C.** Relative mRNA expression of *Pklr, Me1*, *Idh1*, *G6pdx* and *Zeb1* upon siRNA-mediated knockdown of *Zeb1* for 48 h in KPCepi cells (KPC661 and KPC792) by qRT-PCR. *n* = 4 biological replicates. Data are represented as mean ± SEM. ∗*P* < 0.05, ∗∗*P* < 0.01, ∗∗∗*P* < 0.001, ∗∗∗∗*P* < 0.0001 by one-way ANOVA with Tukey’s test (C).

## Data Availability

We have shared the links to data and code in the Materials and Methods section.

## Footnotes

Appendix A Supplementary data to this article can be found online at https://doi.org/10.1016/j.molmet.2026.102382.

## References

[1] Hanahan D., Weinberg R.A. (2011). Hallmarks of cancer: the next generation. Cell.

[2] Pavlova N.N., Thompson C.B. (2016). The emerging hallmarks of cancer metabolism. Cell Metab.

[3] Chambers A.F., Groom A.C., MacDonald I.C. (2002). Dissemination and growth of cancer cells in metastatic sites. Nat Rev Cancer.

[4] Fidler I.J. (2003). The pathogenesis of cancer metastasis: the ‘seed and soil’ hypothesis revisited. Nat Rev Cancer.

[5] Valastyan S., Weinberg R.A. (2011). Tumor metastasis: molecular insights and evolving paradigms. Cell.

[6] Lambert A.W., Pattabiraman D.R., Weinberg R.A. (2017). Emerging biological principles of metastasis. Cell.

[7] Fendt S.M., Frezza C., Erez A. (2020). Targeting metabolic plasticity and flexibility dynamics for cancer therapy. Cancer Discov.

[8] DeBerardinis R.J., Mancuso A., Daikhin E., Nissim I., Yudkoff M., Wehrli S. (2007). Beyond aerobic glycolysis: transformed cells can engage in glutamine metabolism that exceeds the requirement for protein and nucleotide synthesis. Proc Natl Acad Sci U S A.

[9] Beloribi-Djefaflia S., Vasseur S., Guillaumond F. (2016). Lipid metabolic reprogramming in cancer cells. Oncogenesis.

[10] Bergers G., Fendt S.M. (2021). The metabolism of cancer cells during metastasis. Nat Rev Cancer.

[11] Le Gal K., Ibrahim M.X., Wiel C., Sayin V.I., Akula M.K., Karlsson C. (2015). Antioxidants can increase melanoma metastasis in mice. Sci Transl Med.

[12] Piskounova E., Agathocleous M., Murphy M.M., Hu Z., Huddlestun S.E., Zhao Z. (2015). Oxidative stress inhibits distant metastasis by human melanoma cells. Nature.

[13] Tasdogan A., Faubert B., Ramesh V., Ubellacker J.M., Shen B., Solmonson A. (2020). Metabolic heterogeneity confers differences in melanoma metastatic potential. Nature.

[14] Tasdogan A., Ubellacker J.M., Morrison S.J. (2021). Redox regulation in cancer cells during metastasis. Cancer Discov.

[15] Chen T.H., Wang H.C., Chang C.J., Lee S.Y. (2024). Mitochondrial glutathione in cellular redox homeostasis and disease manifestation. Int J Mol Sci.

[16] Seitz R., Tümen D., Kunst C., Heumann P., Schmid S., Kandulski A. (2024). Exploring the thioredoxin system as a therapeutic target in cancer: mechanisms and implications. Antioxidants.

[17] Vanharanta S., Massagué J. (2013). Origins of metastatic traits. Cancer Cell.

[18] Cameron M.D., Schmidt E.E., Kerkvliet N., Nadkarni K.V., Morris V.L., Groom A.C. (2000). Temporal progression of metastasis in lung: cell survival, dormancy, and location dependence of metastatic inefficiency. Cancer Res.

[19] Kienast Y., von Baumgarten L., Fuhrmann M., Klinkert W.E., Goldbrunner R., Herms J. (2010). Real-time imaging reveals the single steps of brain metastasis formation. Nat Med.

[20] Sela Y., Li J., Kuri P., Merrell A.J., Li N., Lengner C. (2021). Dissecting phenotypic transitions in metastatic disease via photoconversion-based isolation. eLife.

[21] Dongre A., Weinberg R.A. (2019). New insights into the mechanisms of epithelial-mesenchymal transition and implications for cancer. Nat Rev Mol Cell Biol.

[22] Yang J., Antin P., Berx G., Blanpain C., Brabletz T., Bronner M. (2020). Guidelines and definitions for research on epithelial-mesenchymal transition. Nat Rev Mol Cell Biol.

[23] Brabletz S., Schuhwerk H., Brabletz T., Stemmler M.P. (2021). Dynamic EMT: a multi-tool for tumor progression. EMBO J.

[24] Schuhwerk H., Brabletz T. (2023). Mutual regulation of TGFβ-induced oncogenic EMT, cell cycle progression and the DDR. Semin Cancer Biol.

[25] Thompson E.W., Redfern A.D., Brabletz S., Berx G., Agarwal V., Ganesh K. (2025). EMT and cancer: what clinicians should know. Nat Rev Clin Oncol.

[26] Stemmler M.P., Eccles R.L., Brabletz S., Brabletz T. (2019). Non-redundant functions of EMT transcription factors. Nat Cell Biol.

[27] Krebs A.M., Mitschke J., Lasierra Losada M., Schmalhofer O., Boerries M., Busch H. (2017). The EMT-activator Zeb1 is a key factor for cell plasticity and promotes metastasis in pancreatic cancer. Nat Cell Biol.

[28] Aurora A.B., Khivansara V., Leach A., Gill J.G., Martin-Sandoval M., Yang C. (2022). Loss of glucose 6-phosphate dehydrogenase function increases oxidative stress and glutaminolysis in metastasizing melanoma cells. Proc Natl Acad Sci U S A.

[29] Hughes C.S., Moggridge S., Müller T., Sorensen P.H., Morin G.B., Krijgsveld J. (2019). Single-pot, solid-phase-enhanced sample preparation for proteomics experiments. Nat Protoc.

[30] Demichev V., Messner C.B., Vernardis S.I., Lilley K.S., Ralser M. (2020). DIA-NN: neural networks and interference correction enable deep proteome coverage in high throughput. Nat Methods.

[31] Subramanian A., Tamayo P., Mootha V.K., Mukherjee S., Ebert B.L., Gillette M.A. (2005). Gene set enrichment analysis: a knowledge-based approach for interpreting genome-wide expression profiles. Proc Natl Acad Sci U S A.

[32] Yu G., Wang L.G., Han Y., He Q.Y. (2012). clusterProfiler: an R package for comparing biological themes among gene clusters. OMICS.

[33] Feldker N., Ferrazzi F., Schuhwerk H., Widholz S.A., Guenther K., Frisch I. (2020). Genome-wide cooperation of EMT transcription factor ZEB1 with YAP and AP-1 in breast cancer. EMBO J.

[34] Balestrieri C., Alfarano G., Milan M., Tosi V., Prosperini E., Nicoli P. (2018). Co-optation of tandem DNA repeats for the maintenance of mesenchymal identity. Cell.

[35] Diaferia G.R., Balestrieri C., Prosperini E., Nicoli P., Spaggiari P., Zerbi A. (2016). Dissection of transcriptional and cis-regulatory control of differentiation in human pancreatic cancer. EMBO J.

[36] Song K.A., Niederst M.J., Lochmann T.L., Hata A.N., Kitai H., Ham J. (2018). Epithelial-to-Mesenchymal transition antagonizes response to targeted therapies in lung cancer by suppressing BIM. Clin Cancer Res.

[37] Davis C.A., Hitz B.C., Sloan C.A., Chan E.T., Davidson J.M., Gabdank I. (2018). The encyclopedia of DNA elements (ENCODE): data portal update. Nucleic Acids Res.

[38] Gertz J., Savic D., Varley K.E., Partridge E.C., Safi A., Jain P. (2013). Distinct properties of cell-type-specific and shared transcription factor binding sites. Mol Cell.

[39] Foggetti A., Baccini G., Arnold P., Schiffelholz T., Wulff P. (2019). Spiny and non-spiny parvalbumin-positive hippocampal interneurons show different plastic properties. Cell Rep.

[40] Prieto Huarcaya S., Drobny A., Marques A.R.A., Di Spiezio A., Dobert J.P., Balta D. (2022). Recombinant pro-CTSD (cathepsin D) enhances SNCA/α-Synuclein degradation in α-Synucleinopathy models. Autophagy.

[41] Segawa M., Wolf D.M., Hultgren N.W., Williams D.S., van der Bliek A.M., Shackelford D.B. (2020). Quantification of cristae architecture reveals time-dependent characteristics of individual mitochondria. Life Sci Alliance.

[42] Schwab A., Rao Z., Zhang J., Gollowitzer A., Siebenkäs K., Bindel N. (2024). Zeb1 mediates EMT/plasticity-associated ferroptosis sensitivity in cancer cells by regulating lipogenic enzyme expression and phospholipid composition. Nat Cell Biol.

[43] Sun X., Peng Y., Zhao J., Xie Z., Lei X., Tang G. (2021). Discovery and development of tumor glycolysis rate-limiting enzyme inhibitors. Bioorg Chem.

[44] Zuo J., Tang J., Lu M., Zhou Z., Li Y., Tian H. (2021). Glycolysis rate-limiting enzymes: novel potential regulators of rheumatoid arthritis pathogenesis. Front Immunol.

[45] Houddane A., Bultot L., Novellasdemunt L., Johanns M., Gueuning M.A., Vertommen D. (2017). Role of Akt/PKB and PFKFB isoenzymes in the control of glycolysis, cell proliferation and protein synthesis in mitogen-stimulated thymocytes. Cell Signal.

[46] Viale A., Pettazzoni P., Lyssiotis C.A., Ying H., Sánchez N., Marchesini M. (2014). Oncogene ablation-resistant pancreatic cancer cells depend on mitochondrial function. Nature.

[47] Choudhury F.K. (2021). Mitochondrial redox metabolism: the epicenter of metabolism during cancer progression. Antioxidants.

[48] Weinberg S.E., Chandel N.S. (2015). Targeting mitochondria metabolism for cancer therapy. Nat Chem Biol.

[49] Fendt S.M. (2019). Metabolic vulnerabilities of metastasizing cancer cells. BMC Biol.

[50] Wei Q., Qian Y., Yu J., Wong C.C. (2020). Metabolic rewiring in the promotion of cancer metastasis: mechanisms and therapeutic implications. Oncogene.

[51] Jiang P., Du W., Mancuso A., Wellen K.E., Yang X. (2013). Reciprocal regulation of p53 and malic enzymes modulates metabolism and senescence. Nature.

[52] Son J., Lyssiotis C.A., Ying H., Wang X., Hua S., Ligorio M. (2013). Glutamine supports pancreatic cancer growth through a KRAS-regulated metabolic pathway. Nature.

[53] Fujiwara-Tani R., Nakashima C., Ohmori H., Fujii K., Luo Y., Sasaki T. (2025). Significance of malic enzyme 1 in cancer: a review. Curr Issues Mol Biol.

[54] Fan J., Ye J., Kamphorst J.J., Shlomi T., Thompson C.B., Rabinowitz J.D. (2014). Quantitative flux analysis reveals folate-dependent NADPH production. Nature.

[55] Wang B., Han X., Lin X., Wang J., You C., Chen K. (2025). The integral membrane protein smim4 modulates redox balance via malate compartmentalization in pancreatic cancer. Nat Commun.

[56] Scherschel M., Niemeier J.O., Jacobs L., Hoffmann M.D.A., Diederich A., Bell C. (2024). A family of NADPH/NADP(+) biosensors reveals in vivo dynamics of central redox metabolism across eukaryotes. Nat Commun.

[57] Sies H., Jones D.P. (2020). Reactive oxygen species (ROS) as pleiotropic physiological signalling agents. Nat Rev Mol Cell Biol.

[58] Ren Y., Mao X., Xu H., Dang Q., Weng S., Zhang Y. (2023). Ferroptosis and EMT: key targets for combating cancer progression and therapy resistance. Cell Mol Life Sci.

[59] Zhang R., Chen J., Wang S., Zhang W., Zheng Q., Cai R. (2023). Ferroptosis in cancer progression. Cells.

[60] Labuschagne C.F., Cheung E.C., Blagih J., Domart M.C., Vousden K.H. (2019). Cell clustering promotes a metabolic switch that supports metastatic colonization. Cell Metab.

[61] Stockwell B.R. (2022). Ferroptosis turns 10: emerging mechanisms, physiological functions, and therapeutic applications. Cell.

[62] Ubellacker J.M., Tasdogan A., Ramesh V., Shen B., Mitchell E.C., Martin-Sandoval M.S. (2020). Lymph protects metastasizing melanoma cells from ferroptosis. Nature.

[63] Nakamura T., Conrad M. (2024). Exploiting ferroptosis vulnerabilities in cancer. Nat Cell Biol.

[64] Biondini M., Lehuédé C., Tabariès S., Annis M.G., Pacis A., Ma E.H. (2024). Metastatic breast cancer cells are metabolically reprogrammed to maintain redox homeostasis during metastasis. Redox Biol.

[65] Benzarti M., Delbrouck C., Neises L., Kiweler N., Meiser J. (2020). Metabolic potential of cancer cells in context of the metastatic Cascade. Cells.

[66] Abbott K.L., Subudhi S., Ferreira R., Gültekin Y., Steinbuch S.C., Munim M.B. (2026). Nutrient requirements of organ-specific metastasis in breast cancer. Nature.

[67] Elia I., Rossi M., Stegen S., Broekaert D., Doglioni G., van Gorsel M. (2019). Breast cancer cells rely on environmental pyruvate to shape the metastatic niche. Nature.

[68] LeBleu V.S., O’Connell J.T., Gonzalez Herrera K.N., Wikman H., Pantel K., Haigis M.C. (2014). PGC-1α mediates mitochondrial biogenesis and oxidative phosphorylation in cancer cells to promote metastasis. Nat Cell Biol.

[69] Yeh H.W., DelGaudio N.L., Uygur B., Millet A., Khan A., Unlu G. (2025). Mitochondrial glutathione import enables breast cancer metastasis via integrated stress response signaling. Cancer Discov.

[70] Youle R.J., van der Bliek A.M. (2012). Mitochondrial fission, fusion, and stress. Science.

[71] Song J., Herrmann J.M., Becker T. (2021). Quality control of the mitochondrial proteome. Nat Rev Mol Cell Biol.

[72] Zhang K., Zhao H., Sheng Y., Chen X., Xu P., Wang J. (2022). Zeb1 sustains hematopoietic stem cell functions by suppressing mitofusin-2-mediated mitochondrial fusion. Cell Death Dis.

[73] Zheng N., Li F., Huang Q., Huang X., Maj T. (2025). Macrophages and macrophage extracellular vesicles confer cancer ferroptosis resistance via PRDX6-mediated mitophagy inhibition. Redox Biol.

[74] Tang D., Chen X., Kang R., Kroemer G. (2021). Ferroptosis: molecular mechanisms and health implications. Cell Res.

[75] Schwab A., Brabletz T. (2025). Grease, fuel and target - polyunsaturated lipids in metastasis. Cell Res.

[76] Viswanathan V.S., Ryan M.J., Dhruv H.D., Gill S., Eichhoff O.M., Seashore-Ludlow B. (2017). Dependency of a therapy-resistant state of cancer cells on a lipid peroxidase pathway. Nature.

