# Corrigendum to “Metabolic plasticity and optimal redox homeostasis are essential for efficient metastatic colonization” [Mol Metab 109 (2026) 102382/42155637]

**DOI:** 10.1016/j.molmet.2026.102401

**Published:** 2026-06-18

**Authors:** Ece Grace, Deyu Zou, Romy Böttcher-Loschinski, Martin Böttcher, Harald Schuhwerk, Yussuf Hajjaj, Annemarie Schwab, Simon Brandt, Ana Clavel Ezquerra, Witold Szymanski, Johannes Graumann, Philipp Arnold, Renato Liguori, Fulvia Ferrazzi, Constantin P. Krempe, Luiza Martins Nascentes Melo, Gabriele Allies, Sven W. Meckelmann, Dirk Mielenz, Simone Brabletz, Dimitrios Mougiakakos, Alpaslan Tasdogan, Thomas Brabletz, Marc P. Stemmler

**Affiliations:** 1 Department of Experimental Medicine 1, Nikolaus-Fiebiger Center for Molecular Medicine, Friedrich-Alexander University of Erlangen-Nürnberg (FAU), Erlangen, Germany; 2 Department of Hematology, Oncology and Cell Therapy, Otto-von-Guericke-University of Magdeburg, Magdeburg, Germany; 3 Magdeburg Centre for Cell and Immune Therapy (MAZI), Medical Faculty, Otto-von-Guericke University, Magdeburg, Germany; 4 Healthcampus Immunology, Inflammation and Infectiology (GC-I3), Medical Faculty, Otto-von-Guericke University, Magdeburg, Germany; 5 Department of Dermatology, University Hospital Regensburg, Regensburg, Germany; 6 Institute of Translational Proteomics & Core Facility Translational Proteomics, Philipps-Universität Marburg, Marburg, Germany; 7 Institute of Functional and Clinical Anatomy, University Hospital Erlangen, Friedrich-Alexander University of Erlangen-Nürnberg (FAU), Erlangen, Germany; 8 Department of Nephropathology, Institute of Pathology, University Hospital Erlangen, Friedrich-Alexander University of Erlangen-Nürnberg (FAU), Erlangen, Germany; 9 Institute of Pathology, University Hospital Erlangen, Friedrich-Alexander University of Erlangen-Nürnberg (FAU), Erlangen, Germany; 10 Comprehensive Cancer Center Erlangen-EMN (CCC ER-EMN), Bavarian Cancer Research Center (BZKF), Erlangen, Germany; 11 Department of Dermatology, University Hospital Essen, German Cancer Consortium (DKTK) and Research Alliance Ruhr, Research Center One Health, Campus Essen, Essen, Germany; 12 Applied Analytical Chemistry, University of Duisburg-Essen, Essen, Germany; 13 Department of Translational Immunology, Department of Internal Medicine 3, University Hospital Erlangen, Friedrich-Alexander University of Erlangen-Nürnberg (FAU), Erlangen, Germany; 14 Center for Health and Medical Prevention - CHAMP, Otto-von-Guericke University, Magdeburg, Germany

The authors regret that the links to raw data, processing and statistical analysis were not included in the published version of the article. The correct version of chapter ‘2.9. Protein mass spectrometric analysis’ in the Materials and methods section should include the following paragraph:

Raw data, processing and statistical analysis can be accessed through the ProteomeXchange Consortium (ProteomeXchange; RRID:SCR_004055) (http://proteomecentral.proteomexchange.org), uploaded via the MassIVE partner mass spectrometry repository with the following identifiers: ProteomeXchange: PXD073617 (https://proteomecentral.proteomexchange.org/cgi/GetDataset?ID=PXD073617), MassIVE ID: MSV000100598 (https://massive.ucsd.edu/ProteoSAFe/dataset.jsp?task=3e48075f61be41468d4c631d06e91e1b).

The authors would like to apologise for any inconvenience caused.

