# Supplementary material for: Metabolic plasticity and optimal redox homeostasis are essential for efficient metastatic colonization
Source: Mol Metab. 2026 May 20;109:102382. doi: 10.1016/j.molmet.2026.102382 (PMC13240752; doi:10.1016/j.molmet.2026.102382)
Supplement: Multimedia component 1 [file mmc1.docx]

**SUPPLEMENTARY METHODS**

**Protein mass spec analysis**

2 × 10^6^ KPCepi cells (KPC661, KPC792, KPC865) and 1.5× 10^6^ KPCmes (KPC550, KPC701, KPC827) and KPCZ (KPCZ346, KPCZ426, KPCZ519) cells were seeded per 10 cm plates and harvested 24h later. Cells were washed three times with PBS, scraped off in 1 ml PBS and collected by centrifugation. The supernatant was removed and the pellet of three replicates snap-frozen in liquid nitrogen. Cell pellets were processed for mass spectrometry (MS)-based proteomic analysis using an adapted single-pot, solid-phase-enhanced sample preparation (SP3) workflow [1] followed by C18 desalting. This approach combines protein solubilization, enzymatic digestion, removal of detergents and desalting into a single streamlined protocol prior to MS analysis. Sample preparation largely followed an in-house procedure (doi.org/10.5281/zenodo.17570947) with minor modifications using 50 µg of protein. All SP3 steps were carried out in 1.5 ml Eppendorf tubes. Peptides were normalized to a final concentration of 100 ng/µl and 300 ng per single injection analyzed by LC–MS/MS on a Bruker timsTOF Pro instrument (Bruker timsTOF Pro mass spectrometer; RRID:SCR_026544). Data acquisition and peptide-spectrum matching were performed using DIA-NN (Data-Independent Acquisition by Neural Networks; version 1.8.1 Academia) [2] following the protocol described in doi.org/10.5281/zenodo.17475274, with the exception that a 100-minute LC method (dia-PASEF - long gradient.m) was employed. Peptide/spectrum matching was performed using a library-free search with a mouse database downloaded from Uniprot.org (RRID: SCR_002380, August 2022, reviewed). A detailed description of the LC-MS method is available at doi.org/10.5281/zenodo.17549946 and complete acquisition parameters may be retrieved directly from the raw data files deposited (see Data and materials availability). Subsequent data handling and statistical evaluation were conducted using the in-house–developed autonomics package (version 1.1.7.15; Bioconductor: 10.18129/B9.bioc.autonomics). Log2-transformed, MaxLFQ [3] values were used for quantification and missing-value imputation. For any given sample, protein group intensities supported by only 1 precursor (Np) were set to NA. Only protein groups passing a q-value threshold of 0.01 were retained for downstream analyses. Differential protein abundance was assessed using the limma framework [4]. All R scripts used for processing the DIA-NN output and for statistical analyses were deposited together with the raw data (see Data and materials availability).

For visualization, heatmaps were generated from the matrix of normalized protein quantification using z-score transformation and the pheatmap R package (v1.0.13). Pathway analysis was performed using Gene Set Enrichment Analysis (GSEA), [5] as implemented in the gseKEGG() function of the clusterProfiler R package (clusterProfiler; RRID:SCR_016884, v4.12.6) [6]. Differentially expressed proteins were ranked according to decreasing log₂-fold change values as input. UniProt protein identifiers (UniProt; RRID:SCR_002380) were mapped to Entrez IDs using the AnnotationDbi (AnnotationDbi; RRID:SCR_023487, v1.66.0) and org.Mm.eg.db (org.Mm.eg.db; RRID:SCR_023488, v3.19.1) R packages. First mapping was used when single Entrez IDs were associated with multiple UniProt identifiers (UniProt; RRID:SCR_002380). KEGG pathway gene sets for the appropriate organism were retrieved internally by the gseKEGG() function with adjusted p-value < 0.05 considered significantly enriched. Data were derived from *n*=9 biological replicates, 3 cell lines for each phenotype/genotype (KPCepi: KPC661, KPC792, KPC865; KPCmes: KPC550, KPC701, KPC827; KPCZ: KPCZ346, KPCZ426, KPC519). For the enrichment analysis, the normalized enrichment score (NES), nominal p-value, and adjusted p-value are reported.

**References**

[1] Hughes, C.S., Moggridge, S., Müller, T., Sorensen, P.H., Morin, G.B., Krijgsveld, J., 2019. Single-pot, solid-phase-enhanced sample preparation for proteomics experiments. Nat Protoc 14(1):68–85.

[2] Demichev, V., Messner, C.B., Vernardis, S.I., Lilley, K.S., Ralser, M., 2020. DIA-NN: neural networks and interference correction enable deep proteome coverage in high throughput. Nat Methods 17(1):41–44.

[3] Cox, J., Hein, M.Y., Luber, C.A., Paron, I., Nagaraj, N., Mann, M., 2014. Accurate proteome-wide label-free quantification by delayed normalization and maximal peptide ratio extraction, termed MaxLFQ. Mol Cell Proteomics 13(9):2513–2526.

[4] Ritchie, M.E., Phipson, B., Wu, D., Hu, Y., Law, C.W., Shi, W., et al., 2015. limma powers differential expression analyses for RNA-sequencing and microarray studies. Nucleic Acids Res 43(7):e47.

[5] Subramanian, A., Tamayo, P., Mootha, V.K., Mukherjee, S., Ebert, B.L., Gillette, M.A., et al., 2005. Gene set enrichment analysis: a knowledge-based approach for interpreting genome-wide expression profiles. Proc Natl Acad Sci U S A 102(43):15545–15550.

[6] Yu, G., Wang, L.G., Han, Y., He, Q.Y., 2012. clusterProfiler: an R package for comparing biological themes among gene clusters. Omics 16(5):284–287.
